# A Review of Senescence‐Associated Secretory Phenotype‐Mediated Remodeling of the Tumor Microenvironment: Implications for Cancer Progression and Therapy

**DOI:** 10.1002/cnr2.70682

**Published:** 2026-09-13

**Authors:** Sneha Pallatt, Sibin Nambidi, Antara Banerjee, Surajit Pathak

**Affiliations:** ^1^ Medical Biotechnology Lab, Faculty of Allied and Healthcare Sciences Chettinad Academy of Research and Education (CARE), Chettinad Hospital and Research Institute (CHRI) Chennai India

**Keywords:** cellular senescence, senescence‐associated secretory phenotype (SASP), senolytics, senomorphics, tumor microenvironment (TME)

## Abstract

**Background:**

Cellular senescence influences cancer progression through irreversible cell‐cycle arrest, immune surveillance, and paracrine signaling mediated by the senescence‐associated secretory phenotype (SASP). SASP comprises inflammatory cytokines, chemokines, growth factors, and matrix‐remodeling enzymes that regulate the tumor microenvironment (TME).

**Objective:**

This review describes the current evidence on the mechanisms of SASP induction, its condition‐dependent roles in cancer, TME remodeling, and emerging strategies targeting senescent cells and SASP signaling.

**Methods:**

Relevant literature was identified through searches on PubMed, Scopus, Web of Science, and Google Scholar using keywords related to cellular senescence, SASP, cancer, TME, senolytics, and senomorphics. Peer‐reviewed English‐language studies published primarily between January 2000‐ March 2026 were considered, including preclinical (in vitro, in vivo), and clinical studies investigating SASP induction and regulation, its role in cancer progression and TME remodeling, and therapeutic strategies targeting senescent cells or SASP signaling.

**Interpretation of the Retrieved Articles:**

SASP exhibits both tumor‐suppressive and tumor‐promoting activities depending on cellular and tissue condition. While transient SASP may enhance antitumor immunity and suppress early tumorigenesis, persistent SASP, particularly during therapy‐induced senescence (TIS), can promote tumor growth, angiogenesis, immune evasion, cancer stemness, metastasis, and therapeutic resistance. Senolytics and senomorphics have therefore emerged as promising approaches for targeting senescent cells and their detrimental secretory effects. SASP‐associated molecules also show potential as biomarkers, although specificity and tissue‐dependent heterogeneity remain major challenges.

**Conclusion:**

Current evidence supports SASP as a major regulator of the TME and an important determinant of cancer progression and therapeutic response. Understanding the condition‐dependent functions of SASP is critical for developing effective senescence‐targeted cancer therapies.

## 1 Background of the Review

Cellular senescence is characterized by permanent cell‐cycle arrest and a distinct secretory phenotype triggered by factors such as telomere dysfunction, oncogene activation, DNA damage, oxidative stress, metabolic stress, and exposure to anticancer drugs [1]. Although senescence functions as an important tumor‐suppressive mechanism by preventing the proliferation of damaged or potentially malignant cells, senescent cells can accumulate in tissues during aging, chronic inflammation, or in response to genotoxic stress and anticancer therapies [2]. A hallmark of senescence is the senescence‐associated secretory phenotype (SASP), a complex mixture of cytokines (e.g., IL‐6, IL‐8), chemokines (e.g., CCL2, CXCL1), growth factors (e.g., VEGF, TGF‐β), proteases (e.g., MMP‐1, MMP‐3), extracellular matrix (ECM) components (e.g., fibronectin, collagen fragments), and extracellular vesicles (EVs) (e.g., exosomes carrying microRNAs and proteins) [3]. These secreted factors can act locally and systemically, enabling senescent cells to influence surrounding tissues and significantly remodel the tumor microenvironment (TME) [3, 4].

Within the TME, SASP‐producing senescent cells play a critical role in regulating tumor‐stroma interactions [4, 5]. During early lesions, temporary senescence and acute inflammatory SASP help the immune system to clear out damaged or transformed cells, which supports tumor suppression [5]. However, persistent senescence, especially therapy‐induced senescence (TIS), arises due to the exposure to genotoxic agents, radiation, targeted therapies, and oxidative stress, leading to chromatin remodeling, mitochondrial dysfunction, and permanent cell‐cycle arrest [6]. This persistent senescent state contributes to chronic inflammation, ECM remodeling, neoangiogenesis, immune suppression, and increased cancer cell plasticity [5, 6]. These effects, together, promote tumor growth, metastasis, and resistance to chemotherapy, radiation therapy, and targeted treatments [6]. Thus, the overall effect of SASP depends on the physiological and pathological conditions and is influenced by factors such as the origin of the senescent cell (whether it is malignant or stromal), the stimulus that caused senescence, genetic background, and the duration of the senescent state [7].

Recognizing the dual and condition‐dependent roles of SASP has sparked interest in senotherapeutics aimed at improving cancer treatment outcomes [7, 8]. Senolytics focus on removing senescent cells, while senomorphics aim to modify or suppress harmful SASP components without killing the cells [8]. These strategies offer hope for reducing the negative remodeling of the TME caused by TIS. Despite growing interest in senescence‐targeted therapies, several translational challenges remain unresolved. Senolytic and senomorphic strategies may cause tissue‐specific effects, unintended immune responses, and toxicity in normal senescent cells that support tissue regeneration and immune functions [9]. In addition, the heterogeneity of SASP composition across tumor types, senescence triggers, and patient populations complicates the development of reliable biomarkers and standardized therapeutic approaches [9]. Therefore, although senotherapeutic interventions show considerable promise in preclinical models, their clinical application in cancer remains largely investigational and requires careful evaluation of safety, specificity, and long‐term outcomes [9].

Although originally recognized as a mechanism that limits the replicative lifespan of cells through telomere dysfunction, senescence is now considered a multifaceted biological program triggered by telomere‐dependent and telomere‐independent pathways [10]. Replicative senescence develops as telomeres progressively shorten and activate a sustained DNA damage response (DDR), while oncogene‐induced senescence (OIS) emerges from aberrant proliferative signaling driven by oncogenes such as *RAS* or *BRAF* [11]. Additional stressors, including metabolic imbalance, chronic inflammation, and mitochondrial or epigenetic perturbations, can also trigger premature senescence [12]. Despite the diversity of these stimuli, senescent cells share core hallmarks, including activation of the p53‐p21 and p16^Ink^ ^4a^/RB pathways, persistent DDR signaling, chromatin alterations, metabolic reprogramming, and enhanced lysosomal activity [12]. These features stabilize the senescent state and influence the secretory profile of senescent cells [12].

SASP composition varies depending on the inducing stimulus, cell type, physiological setting, and microenvironmental factors [13]. Inflammatory SASP components, such as IL‐6, IL‐8, TNF‐α, and CCL family chemokines, drive local and systemic inflammation and shape immune cell recruitment [13]. Pro‐fibrotic and ECM‐remodeling factors, including Transforming growth factor beta (TGF‐β), matrix metalloproteinases (MMPs), and tissue inhibitor of metalloproteinases (TIMPs), alter tissue architecture and promote stromal activation [13, 14]. SASP factors like Vascular Endothelial Growth Factor (VEGF) and Hepatocyte Growth Factor (HGF) support angiogenesis, whereas others, such as WNT ligands, Insulin‐like growth factor‐binding proteins (IGFBPs), and IL‐1α, enhance epithelial‐mesenchymal transition (EMT), stemness, and growth plasticity [14]. Immunomodulatory elements can either promote immune‐mediated clearance of senescent cells or, conversely, create an immunosuppressive niche that fosters tumor progression and therapy resistance [15]. Because SASP influences nearly every component of the TME, it plays a significant role by promoting tumor progression and acts as a potential therapeutic target, making its understanding essential for the development of effective senescence‐based cancer therapies [15].

While there is a current rise in the number of published works about cellular senescence and SASP, the majority of the reviews published so far have either discussed the molecular aspects of senescence or the role of SASP factors on the development of tumors [13, 14, 15, 16]. However, a complete review that combines the time course of SASP development, its microenvironment‐driven role in the TME, and the therapeutic opportunities arising from the complex interplay of these factors remains poorly discussed. For instance, there are limited reviews that have simultaneously discussed the time course of SASP development, its role in the TME, and its application for the development of therapeutic interventions [16]. The current review attempts to address the gap by offering a comprehensive review that combines the molecular aspects of SASP development with its application for the development of senotherapeutic strategies and its role as a biomarker for the development of therapeutic interventions.

### 1.1 Key Advances Highlighted

- Provides an integrated overview from the published literature of SASP‐mediated remodeling of the TME, connecting immune regulation, angiogenesis, fibroblast activation, and ECM remodeling.
- Emphasizes the temporal dynamics of SASP, highlighting how acute versus persistent SASP responses differentially influence tumor suppression or tumor promotion.
- This review discusses emerging evidence linking TIS with chronic SASP signaling that contributes to therapeutic resistance and tumor relapse. It also examines senotherapeutic strategies, including senolytics and senomorphics, and evaluates their potential to modulate SASP‐driven alterations in the TME.
- Finally, this review highlights SASP‐associated molecules as potential biomarkers for monitoring treatment response and guiding personalized therapeutic strategies.

## 2 Methods for Literature Identification

This review focuses on the current literature exploring the role of the SASP in cancer biology and the TME. Relevant studies were identified through searches of major scientific databases, including PubMed, Scopus, Google Scholar, and Web of Science. The search strategy combined keywords such as “cellular senescence,” “senescence‐associated secretory phenotype,” “SASP,” “tumor microenvironment,” “senescence and cancer,” “senolytics,” and “senomorphics.”

The literature search primarily focused on studies published between January 2000 to March 2026, with additional earlier studies included where necessary to provide previous context. Both preclinical studies (in vitro and in vivo) and clinical studies were considered in order to provide a comprehensive overview of the field. Studies were selected based on their relevance to the mechanistic regulation of SASP, its role in tumor‐stromal and immune interactions, and emerging therapeutic strategies targeting senescent cells or SASP signaling. Peer‐reviewed primary research articles and review papers that summarize experimental or clinical evidence were considered for writing this review. Findings were discussed regarding strongly validated mechanisms and studies that demonstrated functional roles of SASP in tumor progression, immune modulation, or therapeutic response.

**FIGURE 1:**
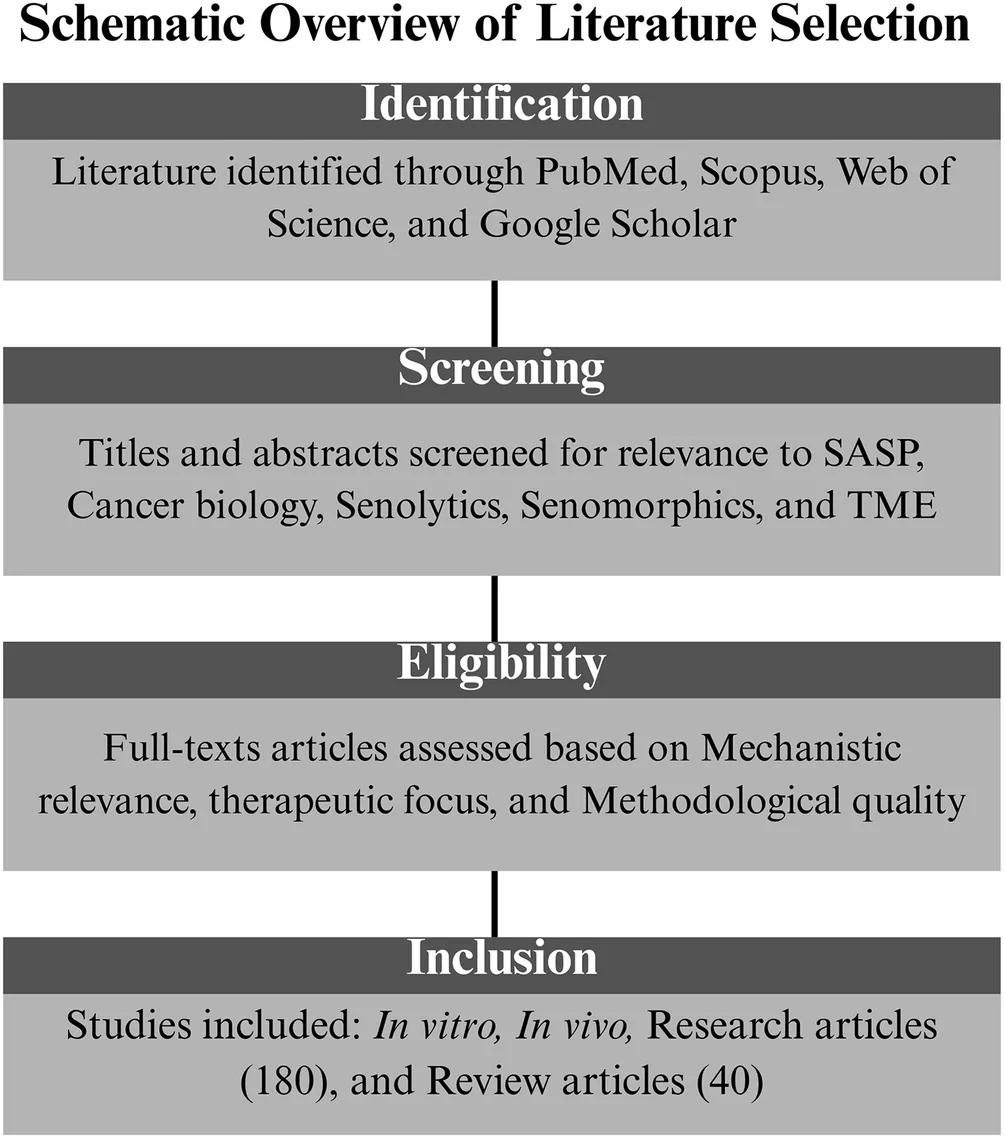
Schematic overview of the literature selection process.

Findings from different studies were interpreted according to experimental model system (preclinical (in vitro, in vivo), or clinical), tumor type, senescence‐inducing stimulus, and stage of clinical translation to distinguish established mechanisms from emerging therapeutic evidence. Because this article presented as a narrative review, a formal systematic quality scoring or meta‐analysis was not performed. Studies were excluded if they were not directly related to SASP function in cancer, focused solely on non‐cancer aging processes, lacked primary experimental or clinical data, editorials, conference abstracts, or contained insufficient methodological information. Only peer‐reviewed articles published in English were considered (Figure 1).

## 3 Molecular Architecture of the SASP

Senescent cells remain viable and metabolically active and undergo profound dysregulation of their transcriptional, translational, and secretory programs [12]. A most important outcome of this cellular reprogramming is the generation of SASP that allows senescent cells to communicate and influence their microenvironment [17]. This provides a clear position of senescent cells as active modulators of tissue homeostasis and disease progression, with particularly significant implications in cancer [18]. Extensive profiling efforts by technologies including antibody arrays, transcriptomics, and proteomics have revealed that the SASP is conserved within specific cell types but varies substantially between them [19]. Thus, senescent fibroblasts, epithelial cells, endothelial cells, and tissue‐resident stromal cells, including stellate cells, all express a robust SASP [19, 20]. However, the exact composition of secreted factors depends on cellular origin and the nature of the applied senescence‐inducing stimuli [20]. These studies uniformly demonstrate that the transition from a proliferative to a senescent state is associated with broad transcriptional reprogramming, particularly involving genes related to cytokine secretion (e.g., *IL6, IL8, CXCL1*), matrix remodeling (e.g., *MMP1, MMP3, MMP9*), metabolism (e.g., *PDK4, GLS1*), and cell‐cycle regulation (e.g., *CDKN2A/p16^Ink4a^, CDKN1A/p21, TP53*) [17, 18, 19, 20]. DNA damage, ROS, and oncogenic stress turn on the p53‐p21 and p16^Ink4a^/RB pathways, establishing cellular senescence and driving the development of the SASP [20, 21].

### 3.1 Core Components and Functional Classes of SASP

The SASP comprises a heterogeneous set of secreted factors that mediate paracrine signaling and proteolytic remodeling of the ECM, enabling senescent cells to influence surrounding tissues [13]. The outcomes of such SASP‐driven signals can be either the support of tissue repair or driving of chronic inflammation, fibrosis, and tumor progression, depending on the cellular and microenvironmental conditions. In cancer, SASP evolves dynamically following senescence induction, with several studies demonstrating a temporal shift in the composition of secreted factors [19]. Experimental evidence indicates that an early SASP phase typically emerges within ~2–4 days after senescence induction, whereas a more pronounced inflammatory SASP becomes evident after ~7–10 days or later, although the precise timing varies depending on the senescence trigger and cell type [22, 23]. Mechanistically, the early SASP is frequently associated with NOTCH‐dependent signaling and is enriched in TGF‐β family members, growth factors, and ECM remodeling proteins [24]. In contrast, the later SASP phase is dominated by NF‐κB‐driven pro‐inflammatory cytokines and chemokines, including IL‐6, IL‐8, IL‐1 family cytokines, and MMPs [18]. This temporal transition from a TGF‐β‐rich early secretome to a pro‐inflammatory cytokine‐dominated SASP has been demonstrated in studies of therapy‐ and oncogene‐induced senescence, where a NOTCH‐regulated early secretory program observed by 3–4 days, transition to an inflammatory SASP by approximately 7–10 days [25].

#### 3.1.1 Cytokines

Among the best‐characterized components, senescent cells secrete a broad range of pro‐inflammatory cytokines, most noticeably IL‐6, IL‐8, TNF‐α, and IL‐1α/IL‐1β, that all together alter the TME and impact cancer initiation, progression, and immune modulation [26]. IL‐6 is among the most abundant and functionally important SASP cytokines [26]. IL‐6 is produced in close association with sustained DNA‐damage signaling in senescent cells independently of *p53* [27]. IL‐6, through IL‐6R/gp130 signaling on surrounding epithelial, endothelial, and stromal cells, drives chronic inflammation, promotes tumor cell survival, and supports malignant growth [28]. In multiple preclinical models including breast, lung, colorectal, and hepatocellular carcinoma, IL‐6 has been shown to promote proliferation, angiogenesis, and metastatic progression [27, 28, 29, 30]. Similarly, IL‐6 has protective or regenerative functions in early stages of liver injury but becomes pro‐tumorigenic in chronically inflamed tissues. Therefore, in SASP‐rich environments, IL‐6 drives pro‐tumorigenic inflammatory TME [26].

Similarly, TNF‐α is a potent SASP cytokine that exerts dual roles in cell death and inflammation. In the TME, the chronic elevation of TNF‐α, often supported by senescent stromal cells, creates conditions that favor tumor growth, immune cell activation, and tissue remodeling [31]. In the study by Micheau and Tschopp (2000), TNF‐α was shown to induce apoptosis in cancer cells primarily through activation of the TNF receptor I pathway, leading to Caspase‐8 mediated cell death [32], persistent TNF‐α‐mediated inflammation generally extends tumor progression and contributes to cancer‐associated cachexia and immune dysregulation [31].

The IL‐1 signaling axis is another central regulator of SASP [33]. Senescent cells markedly upregulate both IL‐1α and IL‐1β, which activate downstream NF‐κB and AP‐1 pathways, leading to transcription of a large fraction of SASP factors [33]. These cytokines are produced by senescent fibroblasts, endothelial cells, and therapy‐induced senescent epithelial cells [32]. IL‐1 signaling does not account for the initiation of senescence but is crucial for the amplification and maintenance of the intensity of SASP [34]. In the TME, IL‐1 cytokines favor tumor growth, immune evasion, angiogenesis, and chronic inflammation. They further act in a paracrine manner to communicate senescence‐like signaling to neighboring cells, causing even greater strengthening of a pro‐tumorigenic environment [34].

#### 3.1.2 Chemokines

Chemokines are a group of small signaling cytokines that facilitate the directed migration of immune cells, and they form a major component of the SASP [35]. Senescent cells secrete a wide range of chemokines, including CXCL1, CXCL2, CXCL5, CXCL8 (IL‐8), CXCL12, CXCL14, CCL2, CCL5, CCL20, and CX3CL1 [35]. The abundant production of these factors establishes a highly inflammatory state within the TME, where they recruit neutrophils, macrophages, lymphocytes, and other immune cells that can unintendedly support tumor growth and progression [36]. Among the CXCL family, CXCL1, CXCL2, and CXCL8 are particularly enriched in the SASP [37]. These chemokines are primarily signaled through the CXCR2 receptor and can create a self‐sustaining inflammatory loop that reinforces senescence through persistent NF‐κB activation [37]. Although this autocrine signaling helps maintain the senescent state, the same chemokines exert strong paracrine influences on surrounding stromal and epithelial cells, promoting cell migration, angiogenesis, and invasive behavior [38]. Senescent fibroblasts and stellate cells also secrete elevated levels of CXCL5 (ENA‐78), CXCL6 (GCP‐2), and CXCL12 (SDF‐1), which contribute further to tumor cell motility, neovascularization, and ECM remodeling [39]. IL8 is especially significant in preclinical tumor models because of its dominant role in neutrophil recruitment and enhancement of tumor invasion and metastatic potential [39]. In vitro and xenograft studies have demonstrated that IL‐8 directly enhances cancer cell invasiveness, angiogenesis, and metastatic behavior [40]. Through its continuous secretion by senescent stromal cells, IL‐8 reinforces a feedback loop of inflammation and promotes cancer cell migration and vascular remodeling within the TME [30]. Senescence also triggers the secretion of numerous CCL family chemokines, such as CCL1, CCL2 (MCP‐1), CCL7 (MCP‐3), CCL8, CCL13, CCL16, CCL20, and CCL26 [39]. These chemokines recruit monocytes, macrophages, and lymphocytes into the TME and help establish a chronic inflammatory environment that facilitates tumor progression [40]. CCL2 and CCL5, in particular, are known to promote the accumulation of tumor‐associated macrophages, a cell population frequently linked to immune suppression, angiogenesis, and enhanced tumor cell survival [39].

#### 3.1.3 Growth Factors

Growth factors represent one of the major functional components of SASP and significantly impact the TME [41]. Among these, senescent cells secrete a broad spectrum of these molecules, including HGF, TGF‐β, Granulocyte‐Macrophage Colony‐Stimulating Factor (GM‐CSF), amphiregulin, epiregulin, Epidermal Growth Factor (EGF), Growth Differentiation Factor 15 (GDF15), and a large group of IGFBPs (IGFBP‐2, −3, −4, −5, −6, and −7) [41]. These secreted proteins regulate proliferation, differentiation, survival, and tissue remodeling. In particular, IGFBPs, which are consistently elevated in senescent fibroblasts and epithelial cells, modulate IGF signaling, thereby influencing tumor cell behavior besides facilitating cellular growth and apoptosis depending upon the cellular conditions [42].

Among these, TGF‐β has a key role by orchestrating both structural remodeling of tissues and paracrine senescence. Early in senescence, a transient NOTCH1 activation increases the secretion of TGF‐β, which, although inducing senescence in bystander cells, transiently inhibits pro‐inflammatory components of SASP [43]. This paracrine reinforcement helps propagate growth arrest beyond the cells receiving the initial damaging signal, but in tumors can be involved in several adverse processes: fibrosis, immune modulation, and resistance to therapy [43].

Evidence from stromal‐cell and xenograft‐based preclinical studies suggests that senescent cells enhance tumor progression through secretion of angiogenic growth factors such as VEGF and Fibroblast Growth Factors (FGFs) [44]. These agents then stimulate the formation of new blood vessels necessary for maintaining continued nutritional and oxygen supply to support both tumor expansion and metastasis. Other regulators like plasminogen activator inhibitor‐1 (PAI‐1) further enhance senescence‐associated signaling by inhibiting cyclin D1 and regulating the ECM turnover [45]. Collectively, active SASP‐associated growth factors may remodel the TME by enhancing angiogenesis, modulating cell‐matrix interactions, and establishing a niche with potentially pro‐tumorigenic features that could promote cancer progression.

#### 3.1.4 Proteases and Other SASP Components

The SASP proteases are a critical structural and functional component of SASP, contributing greatly to the remodeling of the ECM and shaping the TME. The senescent cells frequently secrete MMPs, especially MMP‐2 and MMP‐9, into the medium, degrading the major component of the ECM and promoting tumor cell invasion [46]. As a result of breaking the structural barriers by these MMPs, the tissue architecture gets changed, which provides an environment that can allow cancer cell migration and metastasis [46]. Experimental models developed by Ruhland et al., 2016 [47], have indicated that co‐cultured cancer cells with senescent fibroblasts exhibit enhanced invasive behavior, highlighting the critical role of SASP‐derived proteases in promoting tumor progression [47].

Besides MMPs, SASP also contains several regulatory proteins that modulate ECM and influence tumor dynamics. Tissue inhibitor of metalloproteinases‐2 (TIMP2) is commonly elevated in senescent cells and helps regulate the balance between ECM degradation and synthesis [48]. Though classically an inhibitor of MMPs, dysregulated levels of TIMP2 within the TME lead to disruption of matrix homeostasis and indirectly promote tumor growth. Similarly, PAI‐1, controls fibrinolysis and the stability of ECM by blocking the activation of plasminogen [49].

**FIGURE 2:**
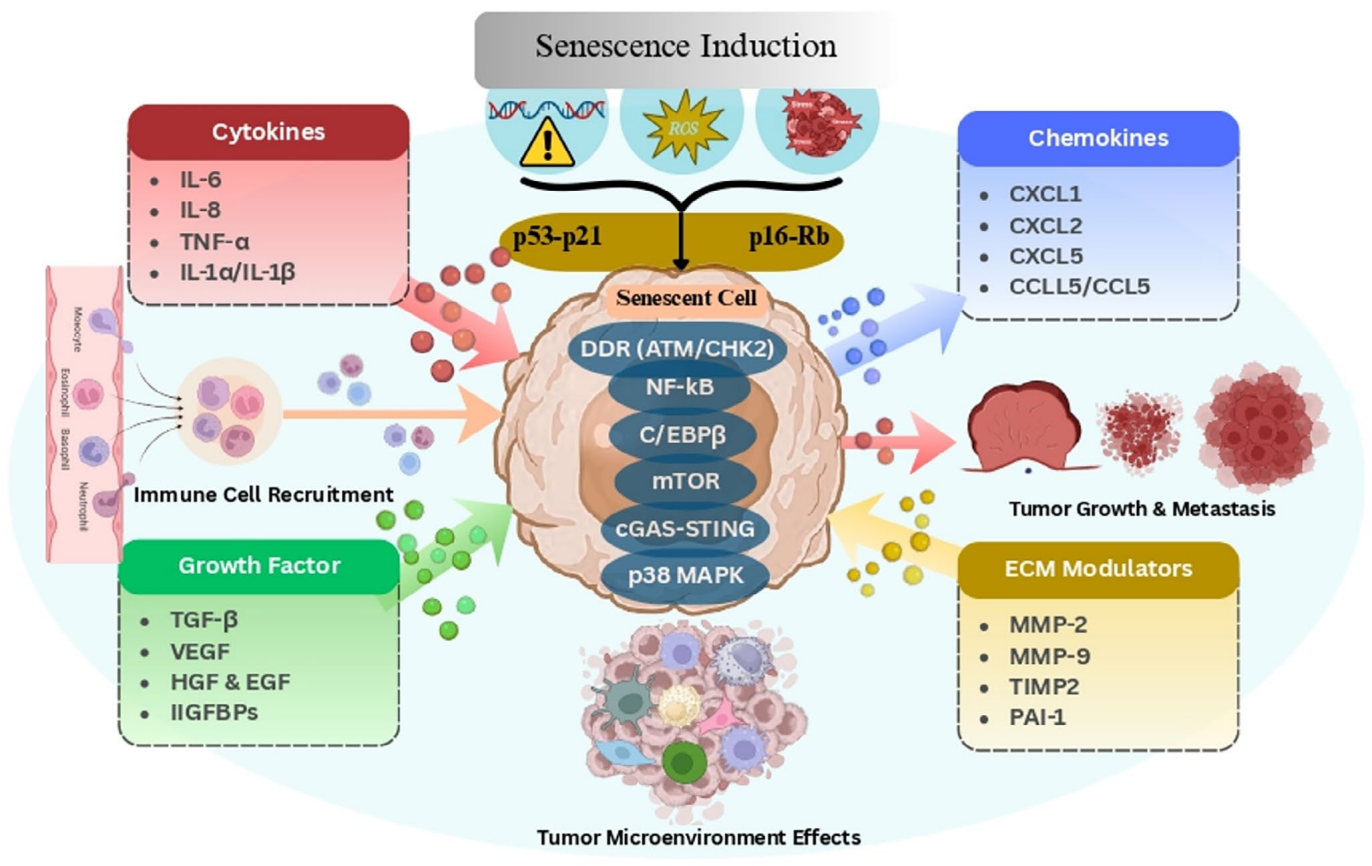
Molecular Architecture and Regulation of the Senescence‐Associated Secretory Phenotype (SASP) [13, 52].

Beyond structural remodeling, SASP‐associated proteases and growth‐related molecules act synergistically to enhance tumorigenesis. The levels of amphiregulin and VEGF are often elevated in senescent fibroblasts, stimulating cancer cell proliferation and angiogenesis, respectively [50]. In murine xenograft and conditioned‐medium experiments, it was demonstrated that tumor cells exposed to SASP‐rich senescent fibroblast secretome formed larger and more vascularized tumors [51]. Altogether, the proteolytic and regulatory components of the SASP play a central role in reshaping the ECM, promoting cancer cell invasion, and creating a microenvironment permissive of tumor development (Figure 2) [52].

#### 3.1.5 Signaling Pathways Driving SASP Induction and Regulation

The SASP is governed by an interconnected signaling network integrating DNA damage response, inflammatory transcriptional reprogramming, innate immune activation, and metabolic control [53]. Rather than being controlled by a single master regulator, SASP emerges from multiple partially overlapping pathways whose contribution varies depending on senescence trigger and cell type [53]. Importantly, mechanistic evidence across models supports distinct levels of involvement, including association, functional necessity, sufficiency, and therapeutic modifiability [53].

The DDR, primarily mediated by ATM/ATR signaling, represents a major upstream event in TIS and OIS [54]. Persistent DDR activation is consistently associated with SASP establishment across irradiation and chemotherapy models [55]. However, DDR signaling alone is not sufficient to induce a complete SASP program, indicating that downstream inflammatory and metabolic pathways are required for full secretory remodeling [56]. Pharmacological or genetic inhibition of DDR components reduces SASP intensity in multiple systems, supporting a role that is condition‐dependent, partially necessary, and therapeutically modifiable [57].

NF‐κB is one of the most robustly implicated regulators of SASP‐associated inflammatory gene expression, including *IL‐6, IL‐8,* and chemokines involved in immune recruitment [56]. Across diverse senescence models, NF‐κB activation strongly correlates with SASP magnitude [56]. Inhibition of upstream components such as IKKβ frequently reduces SASP cytokine production, supporting its role as a functionally necessary pathway for the inflammatory SASP in many, but not all cases [29]. However, NF‐κB activation alone is insufficient to fully recapitulate SASP complexity, indicating that it acts primarily as a transcriptional amplifier rather than an autonomous inducer [29, 56].

The cGAS‐STING pathway links cytoplasmic chromatin fragments arising from genomic instability to innate immune activation and SASP induction [58]. This pathway is particularly relevant in conditions such as TIS and chromosomal instability, where it promotes Type I interferon responses and inflammatory cytokine secretion [59]. Evidence supports its necessity in specific models characterized by nuclear envelope breakdown or cytosolic DNA leakage [59]. However, its role is not universal across all senescence types, and it is insufficient on its own to generate a full SASP program, indicating condition‐specific dependency and partial upstream regulatory function [60].

NOTCH signaling plays a more condition‐specific role, primarily regulating SASP composition rather than acting as a universal driver [61]. In stromal fibroblasts and TME settings, NOTCH activity influences SASP polarity, shifting secretory output toward either inflammatory or matrix‐remodeling phenotypes [61]. While NOTCH signaling can be necessary for specific SASP phenotypic switches, it is not sufficient to induce SASP independently, highlighting its role as a modulatory pathway governing secretory heterogeneity [62].

The mTOR pathway regulates SASP at the level of translation and metabolic control [63]. mTOR activity enhances protein synthesis of SASP‐associated factors, thereby modulating SASP intensity rather than initiating senescence‐associated transcriptional reprogramming [63]. Pharmacological inhibition of mTOR, notably via rapamycin, consistently reduces SASP output across multiple models, indicating partial necessity for full SASP protein expression [64]. However, mTOR is insufficient to induce SASP in the absence of upstream senescence signals, positioning it as a key metabolic regulator of SASP [64].

JAK/STAT signaling contributes primarily to SASP maintenance through autocrine and paracrine cytokine amplification loops, particularly involving IL‐6 family cytokines. This creates a feed‐forward inflammatory circuit that sustains SASP in chronic senescence states [65]. Evidence supports a role for JAK/STAT in maintaining, rather than initiating, SASP expression [66]. Pharmacological inhibition of JAK signaling reduces SASP persistence, confirming its therapeutic modifiability. However, JAK/STAT activation alone is insufficient to induce SASP, reinforcing its role as a downstream amplifying pathway rather than a primary driver [67].

Collectively, SASP regulation is best understood as a hierarchical and condition‐dependent network. DDR and cGAS‐STING function as upstream stress sensors, NF‐κB and NOTCH act as transcriptional and compositional regulators, mTOR governs metabolic and translational output, and JAK/STAT sustains chronic inflammatory reinforcement. Importantly, no single pathway is universally sufficient for SASP induction [54, 55, 56, 57, 58, 59, 60, 61, 62, 63, 64, 65, 66, 67, 68].

### 3.2 SASP Heterogeneity: Determinants of Condition‐Specific Secretory Profiles

The SASP is a very heterogeneous secretory phenotypes that includes soluble factors and EVs released by senescent cells [69]. The pro‐inflammatory SASP, mostly mediated by NF‐κB, underpins inflammaging and makes a substantial contribution to age‐related diseases, such as insulin resistance, tumorigenesis, muscle wasting, and cognitive decline [70]. Importantly, these functional categories are not mutually exclusive. The same SASP can exert overlapping pro‐fibrotic and pro‐inflammatory effects depending on its molecular composition and local microenvironment [71].

The senescence inducer further shapes SASP heterogeneity [72]. OIS, TIS, oxidative stress, and replicative exhaustion all generate unique secretory signatures [72]. For instance, EVs released during OIS promote paracrine senescence in neighboring fibroblasts through the modulation of interferon signaling, whereas EVs released from tumor‐associated senescent cells facilitate the proliferation of cancer cells via EphA2/ephrin‐A1 signaling [72, 73]. Senescent EVs can also carry genomic and mitochondrial DNA that activate innate immune pathways, further diversifying SASP outputs [73]. Such findings recapitulate the emerging view that SASP is not a uniform entity but rather inducer and cell type‐specific secretomes [73].

This heterogeneity arises, at a mechanistic level, from epigenetic remodeling and chromatin reorganization during senescence. Super‐enhancers rich in H3K27ac form key SASP genes, including IL1A and IL1B, allowing for potent transcriptional activation by BRD4 recruitment [73]. At the same time, loss of H3K9me2 permits the expression of pro‐inflammatory cytokines, including IL‐6 and IL‐8 [73]. Three‐dimensional chromatin architecture is also broadly altered during senescence to promote SASP diversity [74]. For example, pericentromeric regions encoding HSATII RNA are converted into a transcriptionally active state, and this HSATII RNA interferes with CTCF in a way that disrupts chromatin looping and favors new regulatory interactions [74]. Therefore, SASP heterogeneity is a product of the integrated actions of cell identity, senescence trigger, temporal state, and epigenetic configuration, which together determine whether SASP acts in a beneficial regenerative role or contributes to chronic inflammation, tissue degeneration, and disease progression.

## 4 SASP and Cancer Hallmarks

### 4.1 Dual Role of SASPs in Cancer

#### 4.1.1 Pro‐Tumorigenic Role of SASP

Cellular senescence is classically recognized as a robust tumor‐suppressive mechanism, initiated in response to oncogene activation, loss of tumor suppressors, oxidative stress and genotoxic therapies [16]. In early tumorigenesis, senescence acts as a barrier that halts the proliferation of transformed cells; for instance, oncogenic RAS or loss of PTEN induces a stable cell cycle arrest accompanied by a SASP [75]. However, when senescent cells persist or accumulate within tissues, their SASP can paradoxically switch from suppressive to pro‐tumorigenic [74, 75]. Several studies, including classical work by Krtolica et al. 2001 [4], showed that conditioned medium from senescent fibroblasts stimulates cancer cell growth through activation of JAK–STAT, MAPK, and NF‐κB pathways, effectively overriding local growth control and enhancing clonogenic expansion [4].

Besides driving proliferation, the SASP factors enable numerous hallmarks of metastatic progression [50]. Pro‐angiogenic mediators such as VEGF, CTGF, and amphiregulin secreted by senescent stromal fibroblasts promote endothelial cell invasion and neovascularization, which can enhance the supply of nutrients and oxygen to tumors [50]. Senescent osteoblasts have been demonstrated in bone metastasis to secrete CXCL12 and other chemokines that facilitate tumor cell adhesion and colonization in the bone microenvironment [76]. Similarly, senescent endothelial cells secrete ECM‐modifying proteins that contribute to the formation of pre‐metastatic niches and thereby facilitate the colonization of distant tissues by tumor cells [77].

In breast cancer and epithelial‐cell experimental models, SASP signaling has been shown to reprogram tumor cell plasticity and promote stem‐like phenotypes associated with relapse and therapeutic resistance [78, 79]. Genotoxic stress‐induced senescence in stromal fibroblasts results in a release of chemokines including IP‐10 (CXCL10) and RANTES (CCL5), enriching tumor‐initiating cell populations and enhancing self‐renewal pathways [50]. IL‐6 and IL‐8 core SASP cytokines have been shown, both in breast cancer models and epithelial models, to drive mammosphere formation, activate EMT‐linked transcriptional reprogramming (Twist, Snail, ZEB1), and upregulate cancer stem cell markers, including CD44 and ALDH1 [80]. Importantly, SASP‐mediated plasticity extends even to non‐transformed cells; exposure of cells to the SASP elaborated by senescent fibroblasts is sufficient to induce partial dedifferentiation or reprogramming‐like states, demonstrating that SASP acts as a broad modulator of tissue plasticity [81].

Beyond tumor‐intrinsic effects, SASP reconfigures tumor‐immune interactions and may establish a permissive, immunosuppressive microenvironment [82]. In various models including TIS in breast and prostate cancers, SASP‐driven MDSC accumulation has been shown to suppress cytotoxic T‐cell and NK cell activity, thus facilitating immune evasion [82]. In the TME, IL‐1α‐driven NF‐κB activation serves as a master amplifier of this self‐reinforcing inflammatory cycle, sustaining SASP production and tissue dysfunction [83].

TIS adds another layer of complexity to treatment outcomes, while chemotherapy or radiotherapy‐induced senescence initially halts tumor proliferation; the accompanying SASP often promotes survival of minimal residual disease [84]. Genotoxic stress‐induced SASP factor, particularly IL‐6, IL‐8, and GM‐CSF, activate pro‐survival signaling, increase drug tolerance, and enrich for stem‐like cancer cells following treatment [85]. In vitro and in vivo breast and colorectal cancer models demonstrated that residual tumor cells exposed to therapy‐induced SASP develop increased resistance to doxorubicin, cisplatin, and radiation [86, 87, 88, 89]. Moreover, senescent tumor cells can eventually escape cell‐cycle arrest, especially when p53, p16, or pRb pathways are compromised, leading to re‐entry into the cell cycle with high genomic instability and aggressive behavior [90].

These findings together illustrate that SASP is a powerful modulator of multiple cancer hallmarks, promoting proliferation, invasion, stemness, immune evasion, genomic instability, and therapy resistance [75, 76, 77, 78, 79, 80, 81]. This duality underlines the therapeutic need to combine senescence‐inducing treatments with strategies that either suppress SASP, neutralize its harmful components, or eliminate senescent cells altogether through senolytics [85, 90]. Such integrated approaches may enable clinicians to exploit the tumor‐suppressive benefits of senescence while minimizing the pro‐tumorigenic consequences of persistent SASP activity.

#### 4.1.2 Anti‐Tumorigenic Role of SASP

Indeed, the SASP plays a key role in tumor suppression during the early stages of tumor initiation [91]. In response to oncogenic activation or stress, senescing cells develop a secretory reprogramming that forms a microenvironment able to block tumor growth by enforcing growth arrest and improving immune‐mediated elimination of damaged or pre‐malignant cells [91]. Senescent cells, through the coordinated secretion of cytokines, chemokines, and proteases, elicit an aggressive anti‐tumor response that stands as one of the major barriers to transformation [92].

A central tumor‐suppressive function of SASP is the activation of immune surveillance [93]. SASP factors such as IL‐1α, IL‐1β, IL‐6, IL‐8, IL‐15, IL‐18, CXCL9, and CXCL10 recruit and activate multiple immune effector populations, including NK cells, CD8^+^ cytotoxic T cells, CD4^+^ helper T cells, macrophages, and dendritic cells (DCs) [93]. NK cells, in particular, represent a crucial player in the elimination of transformed cells. IL‐33 and IL‐1α enhance NK‐cell cytotoxicity through stimulation of IFN‐γ production, while IL‐15, IL‐18, and CXCL10 mediate NK‐cell migration and JAK/STAT‐dependent activation [94, 95]. Comparable SASP‐mediated immune activation has been reported in preclinical liver and pancreatic cancer models, where recruitment of NK and T cells contributes to anti‐tumor immune surveillance, enhanced immune infiltration, and direct cytotoxic elimination of senescent tumor cells [96, 97]. SASP also modulates T‐cell function by promoting CD8^+^ T‐cell cytolytic activity and driving CD4^+^ T‐cell differentiation, and IL‐1 reinforces Th1 polarization to support anti‐tumor immunity [95, 98]. Such T‐cell recruitment by chemokines like CCL2 and CXCL9/10 has already been demonstrated within senescent pancreatic models, highlighting SASP's control over orchestrating early immune defense [99, 100].

Beyond NK and T cells, SASP also regulates the function of macrophages and DCs to further promote tumor elimination. SASP‐associated IFN‐γ and IL‐12 induce M1 macrophage polarization, increasing inflammatory anti‐tumor activity while inhibiting pro‐tumor M2 phenotypes [101]. DCs also become powerful inducers of anti‐tumor immunity under the influence of SASP; IL‐1β promotes DCs to attract IFN‐γ‐producing T cells and clear therapy‐damaged tumor cells, whereas IL‐18 and IL‐33 enhance DC activation and promote NK‐cell‐mediated tumor cell killing [98]. Importantly, SASP can neutralize immunosuppressive cell types in the TME [102]. IL‐33 dampens the suppressive function of MDSCs and limits Treg frequencies, while IL‐12 converts MDSCs into antigen‐presenting cells and reduces Treg‐mediated suppression, altering the immune landscape in a direction that favors improved tumor eradication [102].

Besides promoting immune‐mediated eradication, SASP also directly restrains tumorigenesis by inducing paracrine senescence in adjacent cells [103]. Cytokines like IL‐6, IL‐8, and CXCR2 ligands impose senescence in nearby pre‐malignant cells, thereby restraining their clonal expansion capacity [103]. Such paracrine effects have been elicited in lung cancer (in vitro and in vivo) models, in which NF‐κB activation due to SASP in the neighboring cells triggered senescence and reduced tumorigenesis, and in a prostate cancer model where the mesenchymal stromal cells‐derived SASP induced senescence in immortalized prostate epithelial cells but not in advanced metastatic cells [103]. Indeed, factors such as *PAI‐1* can reverse senescence in fibroblasts, while its overexpression can cell‐cycle exit [104]. SASP components such as IL‐6 and CXCR2 ligands reinforce the irreversibility of senescence, ensuring the potentially oncogenic cells do not re‐enter into the cell cycle [104].

Preclinical Pancreatic Ductal Adenocarcinoma (PDAC) models demonstrated that combined MEK and CDK4/6 inhibition induces senescence‐associated SASP secretion and enhances T‐cell infiltration [105]. In a liver cancer murine model, p53‐driven senescence causes the development of SASP that recruits macrophages, neutrophils, and NK cells for the removal of senescent hepatocytes [106]. The study by Chen et al., 2023 [107], also shows that SASP can associate with IFN‐γ to promote antigen presentation and turn the tumor cells from an immune‐evasive into an immunogenically recognizable state to further promote their elimination through immunosurveillance [107].

**FIGURE 3:**
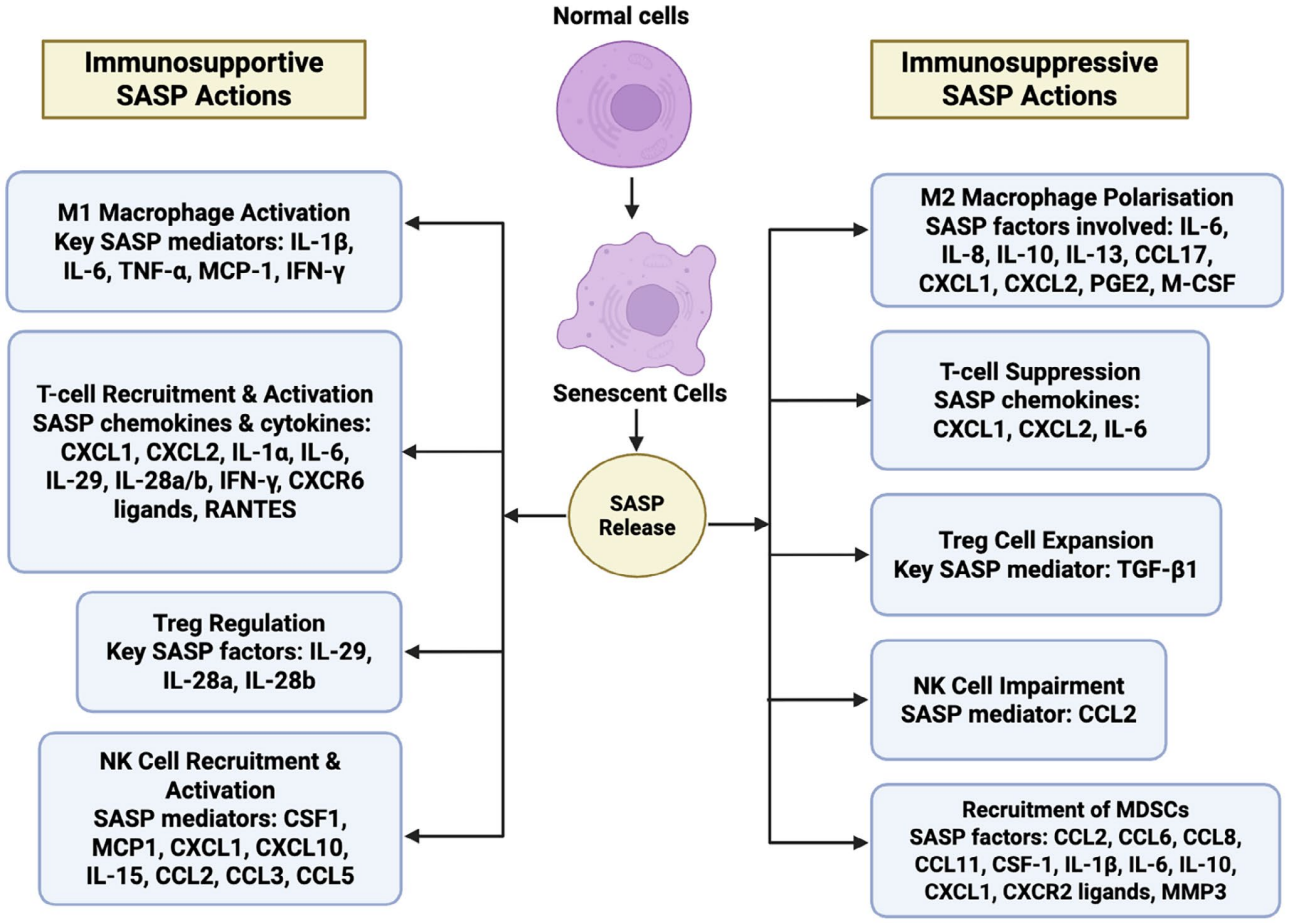
Dual immunomodulatory roles of the senescence‐associated secretory phenotype [107].

These findings emphasize the strong tumor‐suppressive function of SASP in early tumorigenesis. The activation of immune surveillance, the enforcement of paracrine senescence, the stabilization of growth arrest, and the modulation of the balance of immune cell populations all contribute to establishing a microenvironment hostile to the emergence and clonal expansion of malignant cells [104]. Even though in advanced or chronic conditions SASP might drive tumorigenesis, its early anti‐tumor functions remain a cornerstone of senescence‐mediated cancer suppression and an important therapeutic opportunity to improve immune‐mediated tumor control (Figure 3) [107].

### 4.2 SASP and Immune Cell Interaction

SASP modulates the immune landscape of the TME through the regulation of the recruitment, activation, and functional polarization of various immune cell subtypes. Among the best‐characterized interactions are those between SASP factors and macrophages [93, 101]. Senescent tumor cells secrete chemokines and cytokines through pathways such as PI3K/AKT/mTOR, IGF‐1, and IKK2/IκB/p65, driving macrophage infiltration and leading to their polarization toward the pro‐tumorigenic, immune‐suppressive M2 phenotype [108]. TIS or oxidative stress‐induced senescence similarly triggers the release of factors like CXCL2, CXCL3, and IL‐8 that support M2 polarization [109]. However, SASP will not account for M2 differentiation in all instances; in specific conditions, such as p53‐mediated senescence in hepatic stellate cells (HSC) and lung cancer, SASP molecules like IL‐6, IL‐1α, IFN‐γ, and ICAM1 provoke macrophage activation toward the pro‐inflammatory, tumor‐restrictive M1 phenotype [110]. These contrasting observations indicate that macrophage responses to SASP depend on the senescence trigger, tumor type, and local inflammatory milieu [108, 109, 110].

Besides macrophages, equally important are the effects of SASP on T‐lymphocytes [111]. Through chemokines IL‐1α, CCL2, CTACK, RANTES, and CXCL10, senescent tumor cells are capable of attracting CD4+ and CD8+ T cells, enhancing immune surveillance and increasing the efficacy of immune checkpoint blockade (ICB) [111]. Likewise, TIS in pancreatic and melanoma models has been associated with increased expression of VEGF, FGF2, PDGFA/B, and multiple MMPs, facilitating CD8+ T‐cell infiltration into tumors classically termed “cold” in terms of their immunological nature [112]. Very importantly, induction of Types I and II interferons by CDK4/6 inhibitor‐induced senescence increases antigen presentation and improves responsiveness to ICB [113]. On the other side, SASP affects the pool of Tregs: senescent cancer cells are capable of driving the differentiation of Tregs through TGF‐β1, contributing to immune evasion [113]. In some conditions, senescent cell‐derived interferons (IL‐28/29 family) can inhibit Treg proliferation, suggesting that SASP has a condition‐dependent influence on T‐cell subsets [114].

Natural killer T (NKT) cells also respond dynamically to SASP [115]. Senescent osteoblasts induced by ionizing radiation (IR) secrete IL‐6, supporting NKT cell recruitment and a robust inflammatory response, and deletion of either IL‐6 or NKT cells accelerates radiation‐driven osteosarcoma development [115]. In the case of hepatocellular carcinoma models, SASP‐driven CXCR6 signaling enhances NKT cell infiltration in order to contribute to senescent hepatocyte elimination and provide tumor‐suppressive effects [115]. These findings highlight the concept that innate‐like lymphocytes are integral responders to senescence‐associated signals in the TME [114].

Myeloid‐derived suppressor cells (MDSCs) are another immune population that is controlled by SASP. CCL2, IL‐1β, IL‐6, IL‐10, CXCL1, and Colony Stimulating Factor 1 (CSF‐1) secretions by senescent tumor and stromal cells support the recruitment and expansion of MDSCs [116]. These mobilized MDSCs then suppress the cytotoxicity of NK cells and T cells, promote angiogenesis, enhance tumor cell stemness, and contribute to metastatic dissemination [117]. Stromal senescence provoked either by p27 or CDK4/6 inhibitor similarly enhances MDSC infiltration through NF‐κB‐dependent SASP pathways, accelerating tumor growth [117]. Importantly, interference with SASP‐driven chemokine signaling like CXCR2 inhibition has been successful in reducing MDSC recruitment upon chemotherapy‐induced senescence and thus presents a potential therapeutic strategy to counteract senescence‐induced immune suppression [116].

Thus, SASP can enhance anti‐tumor immunity by promoting T‐cell infiltration, antigen presentation, and NKT‐cell‐mediated senescent cell clearance; it can also drive immune evasion through M2 macrophage polarization, Treg expansion, and MDSC recruitment [118]. Importantly, these effects are highly condition‐dependent, influenced by the senescence trigger, tumor type, and local inflammatory milieu. Collectively, SASP acts as a dynamic immunomodulatory network that can either support tumor suppression or facilitate tumor progression [116, 117, 118].

### 4.3 SASP and Stromal Cell Interactions

The SASP of stromal cells within the TME, which play a central role in shaping tumor behavior. Senescent stromal cells, such as fibroblasts, mesothelial cells, and Cancer‐Associated Fibroblasts (CAFs), produce abundant cytokines, chemokines, growth factors, and proteases that remodel tissues and support tumor progression [119]. Coppé et al., 2006 [120] first demonstrated that senescent fibroblasts secrete VEGF, which will drive angiogenesis and accelerate breast cancer progression in immunocompromised mice [120]. Similar effects have been observed across multiple cancer types: senescent mesothelial cells enhance ovarian cancer angiogenic signaling through factors such as CXCL1, CXCL8, HGF, and VEGF, while senescence induced by trametinib plus palbociclib in pancreatic cancer models promotes vascular activation through the secretion of VEGF, PDGF and FGF family members [120, 121]. In HCC, senescent cells release IL‐6, IL‐8, CXCL10, and AREG, which stimulate vascular expansion, although certain regulators such as DNASE1L3 can counteract tumor angiogenesis by reducing SASP output [122].

The senescent stromal cells secrete matrix‐degrading enzymes, as well as pro‐fibrotic mediators which reorganize the main component of the ECM to increase the tissue stiffness, inflammation, and architecture supportive of a tumor [123]. Senescent CAFs are especially powerful, secreting IL‐6, IL‐8, and osteopontin along with other factors associated with resistance to therapy and tumor aggressiveness [124, 125]. Stromal senescence is usually induced by environmental and physiological stresses such as smoking, obesity, aging, and DNA damage due to therapy [125]. Furthermore, aging contributes to the risk of tumors by promoting accumulation of senescent stromal cells whose SASP causes chronic inflammation and dysfunction of the tissue [126].

Obesity‐driven liver cancer explains how SASP elements such as PGE2 suppress anti‐tumor immunity, thus enabling tumor growth [127]. Furthermore, stromal senescence can be enhanced by tumor cells themselves, as cancerous and precancerous cells induce paracrine senescence of neighboring fibroblasts through such chemokines as CXCL1 and GRO‐1, reinforcing a malignant feedforward loop [128]. Thus, SASP‐producing stromal cells are not passive bystanders but rather powerful architects of tumor progression, shaping angiogenesis, ECM integrity, immune regulation, and paracrine signaling to support cancer initiation and expansion (Table 1).

**TABLE 1:** Therapy‐induced senescence (TIS) across tumor and stromal cell types and associated molecular characteristics. IR‐ Ionizing Radiation.

Tumor/tissue origin	Senescence‐inducing therapy	Key senescence markers	SASP/functional characteristics	References
Cancer cells				
Breast cancer	IR/Doxorubicin	SA‐β‐Gal ↑	−/Metabolic recovery phenotype	[86, 87]
Colon cancer	IR	SA‐β‐Gal ↑	Multinucleation and micronuclei formation	[128]
Head and neck	IR/Cisplatin	SA‐β‐Gal ↑, p53 ↑, p21^WAF1^ ↑	CXCL1, CXCL3, CXCL8, CXCL10, CXCL11, IL‐1α, IL‐1β, MIF, VEGF ↑/ IL‐6 ↑, IL‐8 ↑, IL‐1β	[129]
Lung cancer	IR/Neoadjuvant chemotherapy	SA‐β‐Gal ↑	↑ IL‐1α, ↑ IL‐1β	[84, 126]
Lymphoma	Cyclophosphamide + doxorubicin	p53 ↑, p16^Ink4a^ ↑, SA‐β‐Gal ↑	BrdU (−), Ki67 ↓, increased glucose utilization and ATP production	[33]
Melanoma	Cisplatin	SA‐β‐Gal ↑, γ‐H2AX foci ↑, p53 ↑, p21^WAF1^ ↑	IL‐1α, IL‐1β, IL‐6, IL‐8, TNF‐α ↑	[95]
Non‐small cell lung cancer	IR	SA‐β‐Gal ↑	BrdU (−), p53/p21 pathway activation	[28]
Pancreatic cancer	CDK4/6 inhibitor + MEK inhibitor	—	CCL2, CCL4, CCL5, CXCL10, CX3CL1, IL‐15, IL‐18, TNF‐α ↑	[96]
Endothelial cells				
Endothelial cells	Sunitinib	SA‐β‐Gal ↑, p16^Ink4a^ ↑, p53 ↑, p57 ↑	CCL6, complement component C5a, chemerin, IL‐16 ↑	[77]
Fibroblast/CAFs				
Normal human fibroblast	Bleomycin	SA‐β‐Gal ↑, AREG ↑	HGF, IL‐1β, IL‐1α, MCP‐1, MMP‐1, MMP‐2, MMP‐3 ↑	[4, 120]
Human skin fibroblast	IR	SA‐β‐Gal ↑, p16^Ink4a^ ↑, γ‐H2AX foci ↑	SA‐β‐Gal ↑, p16^Ink4a^ ↑, γ‐H2AX foci ↑	[130]
Normal human lung fibroblast	IR	SA‐β‐Gal ↑, p21^WAF1^ ↑, p16^Ink4a^ ↑	—	[131]
CAFs (human lung)	IR	SA‐β‐Gal ↑, 53BP1 foci ↑	Reduced migration and invasion	[119]
CAFs (colorectal cancer)	IR	p53 ↑, p21^WAF1^ ↑	IGF‐1 ↑	[124]
CAFs (HNSCC)	IR	SA‐β‐Gal ↑	BrdU (−), 53BP1 foci ↑	[124]
Immune cells				
Neutrophils (breast cancer)	Chemotherapy	—	Elevated exosomes, nuclear hypersegmentation, metabolic disruption, immunosuppressive phenotype, SIRT1 ↑	[107]
Macrophages (C57BL/6 mice)	IR	SA‐β‐Gal ↑, p16^Ink4a^ ↑, p21^WAF1^ ↑	Elevated exosomes, nuclear hyper‐segmentation, metabolic disruption, immunosuppressive phenotype, SIRT1 ↑	[125]
NK cells (healthy donors)	IR	Morphological changes	CD57 (−), NKG2A ↑	[103]
CD8+ T cells (C57BL/6 mice)	IR	p16^Ink4a^ ↑	Reduced proliferation and metabolism	[132]

### 4.4 Regulation of Extracellular Matrix Dynamics by SASP

There is an extensive structural and biochemical remodeling of the ECM during tumor progression, and SASP factors released from senescent cells play a central role in driving such alterations [130]. Indeed, tumor tissues commonly exhibit increased collagen deposition, with a generally elevated activity of matrix‐remodeling enzymes such as MMPs, LOX/LOXL family members, and WNT‐responsive proteins, which collectively reshape the ECM toward supporting proliferation, invasion, and angiogenesis [133]. Further studies in ovarian, breast, and oral cancers have identified SASP components that include MMP2/10, CXCL10, ITGA2, GBP1, and GATA4 to amplify ECM remodeling and, thereby, promote tumor invasion [134]. Such SASP‐driven ECM cues emerging from senescent myofibroblasts directly fuel EMT in oral submucous fibrosis, a precancerous lesion [134].

A key consequence of SASP activity in cancer is the induction of an inflammatory milieu, which supports excessive ECM deposition, contraction, and stiffening [131]. Enhanced matrix stiffness further promotes tumor growth and metastatic behavior by triggering mechano‐transduction pathways, including integrin‐FAK signaling in HCC, or mechanosensitive cascades in breast cancer driving EMT and therapy resistance [135]. Senescent CAFs further remodel deposited ECM proteins to create stiffer matrices that result in enhanced metastatic dissemination [131]. Importantly, therapies aimed at reducing collagen cross‐linking weaken the tumor stroma and improve T‐cell infiltration, thus enhancing responses to immune checkpoint blockade, which underlines the therapeutic potential of targeting SASP‐dependent changes to the ECM [136].

Senescent stromal cells drive changes to ECM remodeling via not only classical MMP‐mediated degradation but also broader alterations in the levels of soluble and insoluble matrix components [119]. Many reports indicate that chemotherapy, IR, or targeted agents induce TIS that yield stromal cells capable of overproducing MMPs, thus accelerating tumor growth in xenograft models [120]. Beyond MMPs, senescence disrupts the balance of important matrix proteins: for instance, senescent human breast fibroblasts downregulate the tumor‐suppressive proteoglycan decorin while upregulating syndecan‐1, a poor prognostic marker in breast cancer through a paracrine mechanism involving VEGF, bFGF, and TGF‐β [137]. Likewise, the accumulation of ROS associated with senescence activates STAT3 signaling in lung fibroblasts to foster a CAF‐like phenotype that displays heightened collagen contraction and increased cancer cell invasiveness [123]. Other changes to the ECM, such as the glycation of collagen and the subsequent activation of AGE‐RAGE, similarly highlight the integrin‐mediated mechano‐transduction and CAF transformation to promote a TME that fosters tumor development [123].

These findings thus highlight how SASP reshapes the ECM through the cooperative actions of senescent CAFs, MSCs, adipose‐derived stromal cells, and other stromal populations. The resultant ECM dynamics due to SASP drive a permissive niche that facilitates malignant transformation, enhances invasion and metastasis, and modifies therapeutic responses by altering matrix composition, stiffness, and activity of remodeling enzymes. With these extensive insights, precise molecular connections between SASP signaling, fibroblast phenotypes, and ECM remodeling remain incompletely defined and require further investigation to inform stromal‐targeted cancer therapies [123, 137].

### 4.5 SASP‐Induced Inflammatory Microenvironment

In early lesions where p53 remains intact, SASP factors such as IL‐6 and IL‐8 reinforce senescence and help restrain premalignant cells, exemplified by CKIα‐deficient intestinal epithelial cells and RAS‐induced senescent hepatocytes that recruit CCR2^+^ myeloid cells to clear senescent cells through “senescence surveillance” [138]. However, once tumors progress, the same inflammatory signals can become maladaptive; for instance, HCC cells block myeloid cell maturation, converting CCR2^+^ cells into immunosuppressive populations that inhibit NK‐cell‐mediated tumor clearance [138]. Similar condition‐dependent shifts are observed in stromal compartments such as senescent HSCs may promote tumor growth in obesity‐associated NASH‐HCC by responding to pro‐inflammatory stimuli such as deoxycholic acid (DCA), a gut‐derived bile acid, and lipoteichoic acid (LTA), a bacterial cell wall component, leading to increased production of SASP factors including PGE_2_ [114]. In contrast, in cirrhosis‐associated HCC, these cells can reduce fibrosis and support antitumor M1 macrophage polarization [125]. SASP‐driven recruitment of immunosuppressive MDSCs through pathways such as JAK2‐STAT3 further amplifies pro‐tumor inflammation, whereas NF‐κB‐driven SASP programs can promote antitumor immunity [90]. Despite its detrimental roles, SASP can also elicit strong innate and adaptive immune responses under certain conditions; senescent tumor cells often upregulate NK‐cell‐activating ligands (e.g., NKG2D ligands, ICAM‐1), enabling NK‐ or T‐cell‐mediated clearance in lymphoma, multiple myeloma, lung tumors, osteosarcoma, and HCC models [126, 139]. Collectively, these observations reveal that SASP acts as a condition‐dependent dynamic inflammatory network capable of remodeling tissue architecture, modulating immune‐cell recruitment, enhancing angiogenesis, and driving either tumor suppression or tumor progression.

## 5 Tumor‐Type Specific Roles of SASP in the Tumor Microenvironment

Although the core regulatory pathways controlling the SASP are broadly conserved, the functional consequences of SASP signaling in cancer are highly dependent on the physiological and pathological condition. As discussed in the preceding sections, SASP factors can regulate immune cell recruitment, stromal activation, ECM remodeling, and inflammatory signaling within the TME. However, the relative contribution of these mechanisms varies significantly across tumor types due to differences in tissue architecture, stromal composition, immune infiltration, and the nature of senescence‐inducing stimuli.

### 5.1 Breast Cancer

In breast cancer, senescent stromal fibroblasts have been shown to exert profound effects on tumor progression through SASP‐mediated signaling [137]. SASP factors such as IL‐6, IL‐8, VEGF, and MMPs contribute to ECM remodeling, EMT, and enhanced tumor cell motility. These changes can facilitate tumor invasion and metastasis [78, 79]. For example, SASP‐derived cytokines can activate STAT3 and NF‐κB signaling pathways in tumor cells, thereby promoting survival and proliferation despite cytotoxic therapy [87]. Importantly, SASP signaling in breast cancer has also been shown to modulate immune‐cell recruitment, influencing macrophage polarization and inflammatory responses within the TME [79, 86, 87].

### 5.2 Colorectal Cancer

In colorectal cancer, SASP signaling plays an important role in shaping the inflammatory microenvironment of the intestinal epithelium [40]. Senescent cells within colorectal tumors or surrounding stromal compartments can secrete cytokines such as IL‐6, IL‐8, CXCL1, and TGF‐β, which influence immune‐cell recruitment, angiogenesis, and tumor cell survival [128]. Because the intestinal mucosa is already enriched in immune cells and microbial‐derived inflammatory stimuli, SASP signaling may amplify inflammatory signaling pathways that promote tumor progression [140].

SASP‐mediated cytokine signaling can activate STAT3 and MAPK pathways, thereby promoting tumor cell proliferation and survival [141]. However, senescence also plays tumor‐suppressive roles in colorectal cancer, particularly in early tumorigenesis where OIS can limit the expansion of transformed cells [141]. Persistent or unresolved senescence, may shift the balance toward chronic inflammation and tumor‐promoting signaling [141].

### 5.3 Lung Cancer

In lung cancer, TIS has been observed following exposure to chemotherapy, radiation therapy, and targeted agents [84]. Senescent lung tumor cells and stromal cells can release SASP factors including IL‐6, IL‐1β, CXCL chemokines, and TGF‐β, which influence immune responses and inflammatory signaling in the lung microenvironment [39]. These mediators can promote tumor cell survival and stimulate angiogenesis through paracrine signaling [28, 39]. Importantly, SASP signaling in lung cancer may exert dual effects depending on the condition. In early stages, SASP‐associated chemokines can recruit immune cells that contribute to the clearance of senescent tumor cells [126]. However, persistent SASP signaling may create a pro‐tumorigenic inflammatory environment that supports tumor recurrence and progression [142].

### 5.4 Pancreatic Cancer

PDAC is characterized by a dense fibrotic stroma and a highly immunosuppressive TME [96]. Senescence‐associated secretory signaling has been implicated in shaping this unique tumor niche [96]. SASP factors derived from senescent stromal fibroblasts or pancreatic stellate cells can promote ECM deposition, fibrotic remodeling, and immune suppression, all of which contribute to the aggressive nature of pancreatic tumors [100, 105]. Cytokines such as IL‐6, IL‐8, and TGF‐β, as well as growth factors and matrix‐remodeling enzymes, can enhance tumor cell proliferation and support metabolic adaptation within the nutrient‐poor pancreatic TME [105]. Moreover, SASP‐mediated inflammatory signaling may contribute to resistance to chemotherapy and targeted therapies by activating survival pathways in tumor cells and by modifying immune‐cell infiltration within the tumor stroma [105].

These studies collectively suggest that, although the molecular regulators of SASP are broadly conserved, the functional consequences of SASP signaling are strongly influenced by tumor‐specific microenvironmental factors, including stromal composition, immune infiltration, and the nature of senescence‐inducing stimuli [105]. A deeper understanding of these tumor‐type specific differences will be important for developing therapeutic strategies that either eliminate senescent cells or selectively modulate SASP signaling in cancer.

## 6 SASP in Cancer Therapy and Treatment Response

### 6.1 Therapy‐Induced Senescence (TIS)

TIS is now considered one of the major cellular responses to conventional anticancer therapies like chemotherapy, radiotherapy, and certain targeted therapies [78]. These interventions cause significant DNA damage and cellular stress on both malignant and non‐malignant cells within the TME, driving them into a senescent state characterized by stable cell‐cycle arrest [143]. Both preclinical investigations and limited clinical observations indicate that features of TIS are evident in 31%–66% of tumors following chemotherapy, and senescent cells can also be present in surrounding normal tissues following chemo‐ or radiotherapy [144]. Whereas senescence was once thought to be a desirable outcome of cancer therapy because it arrests proliferation, it is now recognized that TIS has dual effects: while it may initially delay tumor growth, it may also establish a microenvironment that promotes tumor recurrence, metastasis, or therapeutic resistance [144, 145] (Table 2).

**TABLE 2:** Condition‐dependent framework of SASP: Differential tumor‐suppressive and tumor‐promoting consequences based on senescence type, cellular origin, and temporal persistence. TIS‐ Therapy‐induced senescence, OIS‐ Oncogene‐induced senescence.

SASP context	Senescence type	Cellular source	Duration	Dominant SASP output	Biological outcome	References
Early tumorigenesis	OIS	Premalignant tumor cells	Acute	IL‐6, IL‐8 (transient), immune‐attracting cytokines	Tumor suppression via immune clearance	[75, 104]
Early therapy response	TIS	Tumor cells	Acute	Pro‐inflammatory SASP, chemokines	Tumor regression + immune recruitment	[56]
Persistent tumor senescence	TIS	Tumor cells	Chronic	IL‐6, IL‐8, CXCLs, TGF‐β	Therapy resistance, relapse, stemness	[6, 141]
Stromal senescence	Therapy‐ or stress‐induced	Fibroblasts/CAFs	Chronic	MMPs, HGF, IL‐1β, IL‐6	ECM remodeling, invasion, metastasis	[117, 125]
Endothelial senescence	Stress/therapy‐induced	Endothelial cells	Chronic	VEGF, chemokines, inflammatory mediators	Angiogenesis, tumor vascular remodeling	[32, 77]
Immune cell senescence	Therapy‐induced/tumor‐driven	T cells, NK cells, macrophages	Chronic	Immunosuppressive cytokines, altered metabolism	Immune evasion, reduced cytotoxicity	[143, 146]

A broad spectrum of chemotherapeutic agents induces senescence in tumor cells as well as in the surrounding stromal cells [144]. Topoisomerase inhibitors, such as etoposide and camptothecin, induce DNA double‐strand breaks and are established models for the induction of senescence in primary and immortalized cancer cells [147]. Alkylating agents include doxorubicin, melphalan, and cyclophosphamide, which, in turn, similarly activate senescence pathways in various cell types [86]. Doxorubicin, for example, intercalates into DNA and interferes with topoisomerase‐II‐mediated repair processes, thus inducing senescence in fibroblasts and cancer cells [86, 87]. Other chemotherapeutic agents, such as temozolomide in glioblastoma and cisplatin in ovarian, lung, liver, and nasopharyngeal cancers, induce senescent phenotypes [129]. Whether chemotherapy causes senescence or apoptosis depends on the drug dosage, duration, and functional status of senescence‐associated regulators such as p53 and p16 [143]. Generally, high drug concentrations tend to favor apoptotic cell death, whereas intermediate levels tend to cause senescence [143].

Radiotherapy is another potent inducer of TIS and causes widespread DNA damage through IR [143]. Irradiated cells are characterized by typical senescence markers such as SA‐β‐gal activity, *p53* and *p21* upregulation, and increased SASP factor production [132]. Radiation‐induced senescence has been described in brain, breast, and lung cancer, and ablation of radiation‐senescent cells in the brain microenvironment has been demonstrated to decrease glioblastoma recurrence [132]. In contrast to chemotherapy, radiotherapy often causes localized but persistent senescence in surrounding healthy tissues, aggravating its clinical consequences [148].

Targeted therapies can also induce senescence by impairing key oncogenic pathways; inhibitors targeting  CDK, NOTCH, CK2, MDM2, JAK2, and SKIP2 are associated with growth arrest and senescence in various tumor models [149]. Among others, the CDK4/6 inhibitor palbociclib constitutes one of the most active senescence inducers known thus far, inducing robust senescent phenotypes in triple‐negative breast cancer [150]. Combinatorial interventions, including combination treatments involving CDK4/6 or aurora kinase A inhibitors with MDM2 inhibitors, can potentiate senescence, leading to an improved SASP‐driven recruitment of cytotoxic T‐cells and better immune‐mediated eradication of senescent tumor cells [121, 151]. HER2‐targeting therapy with lapatinib similarly induced a senescence‐like appearance that was supported by increased expression levels of p15 and p27 and by the over‐expression of SA‐β‐gal activity [152]. While this cell‐intrinsic form of TIS may contribute to early drug efficacy, it could also be engaged as part of adaptive resistance mechanisms [153]. Other molecules, including the CDK2 inhibitor INX‐315, further support the idea that exploiting pathways involved in TIS may form the rationale for novel senescence‐oriented cancer therapies [153].

Distinct therapeutic interventions generate heterogeneous senescence reprogramming with differences in the stability of growth arrest, temporal dynamics, and qualitative composition of the SASP [154]. In addition to treatment‐related variables, host factors such as age critically influence the induction and persistence of senescence [153, 154]. Moreover, age‐associated changes may amplify pro‐tumorigenic effects of the SASP, including immune suppression, ECM remodeling, and metastatic niche formation under certain conditions [18]. Therefore, the biological and clinical consequences of TIS are highly condition‐dependent and may differ substantially across cancer types, therapeutic regimens, and patient populations. However, the biological outcomes of TIS are condition‐dependent, and its clinical implications remain complex. While TIS can contribute to tumor suppression, persistent senescent cells and sustained SASP signaling may also promote tumor recurrence, immune dysregulation, and therapy‐related toxicity. Therefore, therapeutic strategies targeting TIS require careful consideration of tumor type, treatment regimen, and patient‐specific factors [154].

### 6.2 SASP‐Driven Regulation of Immunotherapy Response

SASP actively promotes the chronic, low‐grade inflammation characteristic of aging, also known as inflammaging, and this state strongly controls how the immune system will respond to cancer and especially immunotherapy [155]. Nguyen and Cho (2025) [156], point out that chronic SASP from a growing senescent burden accelerates systemic inflammation and immune dysfunction, both key elements of immune‐senescence [156]. This pro‐inflammatory environment seeks to develop a paradoxical effect by impairing the efficiency of immunotherapies while it aims to recruit immune surveillance [156]. On one hand, SASP factors such as IL‐6, IL‐1β, and other cytokines signal through NF‐κB, JAK/STAT, along with other inflammatory pathways that reinforce immune activation and potentially improve antigen presentation [157]. In contrast, aged individuals or those experiencing chronic inflammation, this same SASP‐driven inflammation can drive immunosuppressive response [158]. As immune‐senescence progresses, the crucial immune cell populations, such as T cells and antigen‐presenting cells, will undergo functional deterioration, diversity reduction, and proliferative potential loss [158].

In this way, SASP contributes to a TME that attracts immune cells while blunting their anti‐tumor activity, undermining the durability of cancer immunotherapy. Moreover, SASP‐induced inflammaging may favor the expansion of suppressive immune subsets [157]. For example, chronic exposure to pro‐inflammatory SASP cytokines supports the persistence of MDSCs and Tregs that dampen cytotoxic T lymphocyte responses [159]. This immunosuppressive shift decreases the therapeutic efficacy of immune checkpoint inhibitors by limiting the infiltration and function of effector T cells [159]. Simultaneously, immunotherapy may be influenced by immune senescence: with the aging of the immune function, ICI or CAR‐T cells may be less effective because of a decrease in T‐cell diversity, together with increased exhaustion and reduced proliferative capacity [160]. Thus, SASP is not simply a passive inflammatory background; it reshapes actively the immune microenvironment and limits therapeutic potency. Importantly, targeting SASP or more broadly, limiting inflammaging can strengthen immunotherapy efficacy [160].

The strategies may include senolytic drugs to remove senescent cells, senomorphic agents to suppress harmful SASP factors, or immune‐rejuvenation therapies, a checkpoint modulator, TLR agonist, or metabolic regulator to reconstitute the immune function [160]. Such combination approaches could improve T‐cell responsiveness and reduce immunosuppressive cell populations by lowering the chronic inflammatory burden due to SASP, thereby leading to improved outcomes with ICIs or adoptive cell therapies [161]. The dual role of SASP in both immune activation and immune suppression highlights the need for careful translational validation before therapeutic targeting in clinical oncology [162].

### 6.3 Senescence Escape and Its Implications in Cancer Progression

Although cellular senescence is traditionally viewed as an irreversible growth‐arrested state, growing evidence demonstrates that a portion of senescent tumor cells can re‐enter the cell cycle, referred to as senescence escape [163]. Importantly, these traits are restricted to a minority of senescent cells and are strongly influenced by cellular state, particularly the integrity of key tumor suppressor pathways, including p53 and p16^Ink4a^ [1, 2]. Detecting senescence escape requires stringent approaches such as single‐cell tracking or lineage tracing to exclude expansion of pre‐existing non‐senescent clones [164]. Nonetheless, experimental data support the fact that senescent cells, upon persistent stress, chromatin remodeling, or checkpoint attenuation, are able to restart their proliferation and form genetically and phenotypically heterogeneous progeny [164].

These senescent escaped cells often develop aggressive phenotypes, which include increased clonogenicity, cancer stem cell‐like properties, EMT, and general resistance to therapy [165]. These characteristics reflect the profound reprogramming that occurs during chronic senescence, often with accompanying DNA damage accumulation, chromosomal instability, and epigenetic changes [165]. In cancer therapy, TIS represents a potentially concerning mechanism of tumor cell escape [166]. Whereas treatment at the outset drives damaged cancer cells into arrest, subsets persist long‐term and eventually break through senescence checkpoints via mutations, loss of p53/p21 or p16/Rb signaling, polyploidy‐driven genome reshuffling, or epigenetic reprogramming [166]. These escaped cell populations have been implicated in tumor recurrence, residual disease maintenance, and metastatic progression [166].

The SASP itself can facilitate the escape. Chronic SASP signaling, in particular through IL‐6/STAT3, IL‐8/CXCR, TGF‐β, WNT, and IGF pathways, creates a microenvironment rich in inflammatory, mitogenic, and pro‐survival cues [166]. These cues can weaken cell‐cycle checkpoints within senescent tumor cells, support metabolic reprogramming, and enhance stemness features, a general way to promote conditions favorable for cell‐cycle re‐entry [167]. SASP‐driven autocrine and paracrine signaling can also impose additional DNA damage and oxidative stress, further destabilize the senescent state and promote the escape [167]. Thus, senescence escape represents a paradoxical outcome of a normally protective tumor‐suppressive response. When senescent cells persist rather than being cleared, they become a source of highly malignant, therapy‐resistant progeny [167].

### 6.4 Beneficial Roles of the SASP in Tissue Repair and Tumor Suppression

Acute SASP generated during TIS represents a potent, time‐restricted immune‐activating program that significantly enhances the anti‐tumor effects of chemotherapy, radiotherapy, and targeted therapies [143]. This beneficial role is underscored by the in vivo studies of (Liu et al.,2013): senescence induced by aurora kinase A or CDK4/6 inhibition in melanoma leads to a chemokine‐rich SASP that promotes dense infiltration of CD4^+^ and CD8^+^ T cells, resulting in marked tumor growth suppression [151]. Similarly, chemotherapy‐induced senescence in lymphoma demonstrates that NF‐κB‐dependent SASP is essential for NK‐cell recruitment and therapeutic efficacy, as inhibition of SASP markedly diminishes treatment benefit [146]. Together, these studies show that acute SASP functions as a biological “adjuvant,” coupling senescence arrest to robust immune‐mediated tumor clearance [146].

**FIGURE 4:**
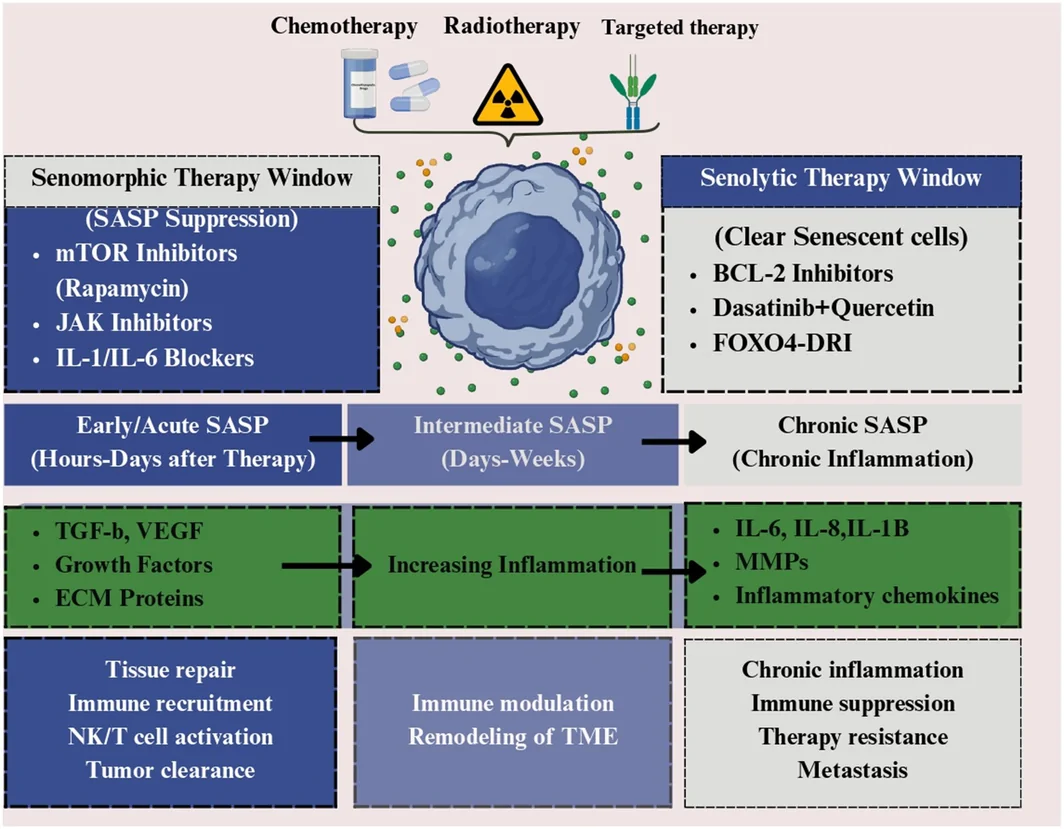
Temporal dynamics of SASP during cancer therapy and therapeutic targeting window [146].

However, the effects of SASP depend largely on its duration and timing, with beneficial effects occurring primarily during the early stages of senescence. In other words, acute SASP acts like a potent, self‐limiting immunogenic signal that enhances senescence‐based therapies by allowing for the immune elimination of damaged cells, while chronic SASP cancels this effect and promotes tumor persistence and recurrence (Figure 4) [146].

## 7 Therapeutic Targeting of the SASP in Cancer Treatment

### 7.1 Senolytics

**FIGURE 5:**
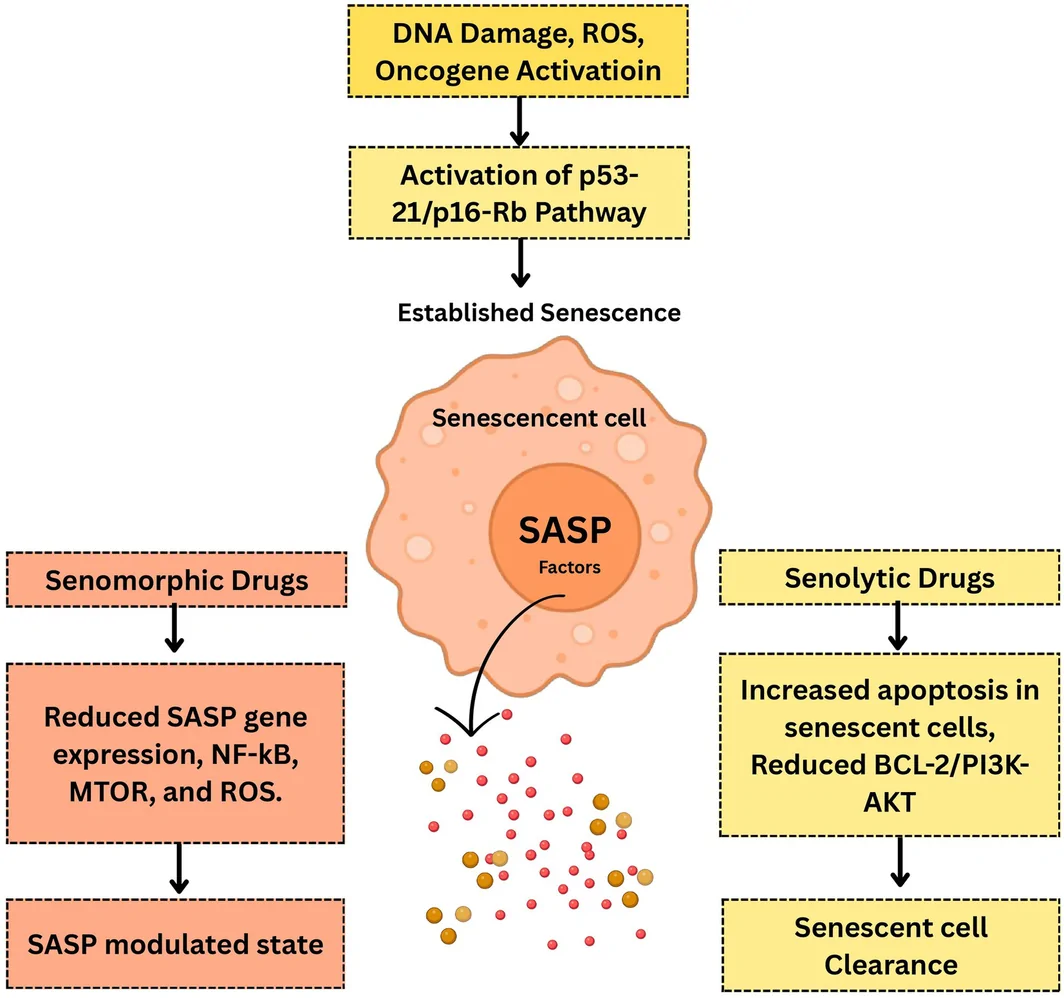
Therapeutic targeting of senescent cells and the SASP in cancer [169].

They represent a promising strategy to eliminate TIS cells and mitigate the detriments associated with chronic SASP signaling within the TME [168]. In contrast to proliferating tumor cells, senescent cells acquire resistance to apoptosis through upregulation of several anti‐apoptotic pathways that include BCL‐2 family signaling, PI3K/AKT, and p53‐FOXO4 interactions [8, 9]. Senolytic drugs leverage these characteristics, inducing programmed cell death selectively in senescent cells and thus dampening SASP‐driven inflammation, fibrosis, neo‐angiogenesis, and finally therapy resistance [168]. Removing residuating senescent cells subsequent to chemotherapy, radiotherapy, or targeted therapy, senolytics confine not only tumor relapse but also improve the effectiveness of conventional and immunotherapeutic interventions (Figure 5) [170].

Of the senolytics under current investigation, BCL‐2 family inhibitors are the most extensively studied class [8]. Preclinical studies across ovarian, breast, lung, pancreatic, and hematologic cancer models have demonstrated that Navitoclax (ABT‐263) and its precursor ABT‐737 effectively induce apoptosis in senescent tumor cells as well as senescent stromal components [171]. Similarly, obatoclax, a BH3 mimetic, enhances the response to bromodomain inhibitors in triple‐negative breast cancer and extends survival in diffuse intrinsic pontine glioma by targeting BMI1‐driven senescence programs [172]. These findings emphasize the clinical relevance of inhibiting senescent cell specific survival pathways in a wide range of malignancies.

Natural and synthetic small molecule senolytics also have emerged as promising therapeutic agents [173]. One such combination, dasatinib and quercetin, has shown extensive senolytic coverage, removing senescent fibroblasts, CAFs, and senescent tumor cells in preclinical models [173]. Fisetin, a quercetin‐like flavonoid, provides similar enhancements to tumor cell apoptosis and has been utilized to delay age‐associated and cancer‐related phenotypes in mice [174]. Procyanidin C1, a polyphenolic compound, bifunctionally induces apoptosis in senescent cells while repressing SASP expression, further contributing to tumor regression and reduced chemoresistance in prostate cancer [175]. Other senolytic modalities, such as ouabain, which targets Na^+^/K^+^‐ATPase, and the FOXO4‐DRI peptide, which disrupts FOXO4‐p53 survival signaling in senescent cells, continue to build on mechanistic diversity for senolytic strategies [176].

The drug‐delivery systems, including galacto‐oligosaccharide encapsulation, lipofuscin‐targeted dasatinib conjugates, and PEO‐b‐PCL micelles, dramatically diminish systemic toxicity with a pronounced increase in senescent cell targeting [177]. Ferroptosis‐based strategies and iron‐responsive prodrugs represent promising approaches for eliminating both primary senescent cells and secondary senescent populations generated through paracrine SASP signaling [178]. These advances overcome key limitations of the first generation of senolytics that were associated with thrombocytopenia and neutropenia upon Navitoclax administration and enhance the translational potential of senolytic interventions [179].

However, despite their promise, senolytic therapies must be applied cautiously, as senescent cells also possess condition‐dependent physiological roles including tissue repair and endocrine support [180]. Further, SASP itself can be beneficial in the acute phase of senescence, for example by stimulating immune clearance of damaged cells and enhancing antitumor immunity [180]. Consequently, the timing of senolytic administration relative to senescence induction is paramount [181]. Complementary strategies include senostatics, which aim to neutralize harmful SASP factors without killing senescent cells. Examples of senostatics include metformin (an mTOR/STAT3 inhibitor), sertraline, which suppresses SASP by inhibiting mTOR, and approaches targeting regulators such as PTBP1 that selectively block pro‐tumorigenic SASP components without abrogating growth arrest [181, 182].

However, the biological outcomes of TIS are highly condition‐dependent, and its clinical implications remain complex. While TIS can contribute to tumor suppression, persistent senescent cells and sustained SASP signaling may also promote tumor recurrence, immune dysregulation, and therapy‐related toxicity. Therefore, therapeutic strategies targeting TIS require careful consideration of tumor type, treatment regimen, and patient‐specific factors [182].

### 7.2 Senomorphics

While senolytics induce apoptosis of senescent cells, senomorphics suppress or reshape the SASP to minimize its inflammatory and tissue‐destructive functions [183]. This approach is especially useful in settings where senescent cells continue to perform useful functions, such as wound healing, resolution of fibrosis, and immune surveillance [183, 184]. By dampening pathological SASP outputs without disrupting growth arrest, senomorphics provide a more discriminating and physiologically compatible approach to treatment of senescence‐driven dysfunction in cancer and aging [184].

The mTOR inhibitors like rapamycin, which suppress the production of SASP by limiting the translation of MK2 kinase through 4EBP1 and by reducing the IL‐1α‐dependent upstream signaling [185]. Rapamycin has also been shown to mitigate the growth‐promoting effects of senescent fibroblasts on tumors, but also improves tissue homeostasis and stem cell function in aging models [185]. Hypoxia mimetics like roxadustat likewise dampened SASP output, but enhanced resilience in chemotherapy‐treated and old mice, reinforcing the therapeutic potential of SASP modulation [186].

Other inhibitors of the p38‐MK2 axis include CD111 and ATI‐450, which have exhibited potent senomorphic effects through the suppression of SASP‐associated inflammatory mediators and the metastatic spread in models of breast cancer [187]. Similarly, inhibitors of the JAK pathway, such as NVP‐BSK805 and clinically used agents including ruxolitinib and baricitinib, blunt IL‐6/IL‐8‐driven downstream inflammatory signaling and improve tissue repair, hematopoietic function, and therapeutic responses in cancer models [188].

Apart from targeting upstream signaling pathways, senomorphics also include biologics neutralizing specific SASP factors or their receptors. IL‐1 receptor antagonists, such as anakinra, inhibit SASP‐mediated invasiveness through the down‐regulation of IL‐6 and IL‐8 secretion [189]. Agents targeting TNF‐α, such as adalimumab, can mitigate the SASP‐driven inflammation and associated tumor‐promoting effects [189]. Monoclonal antibodies against either IL‐6 (siltuximab) or IL‐6R (tocilizumab) are receiving increasing attention as selective modulators of SASP‐driven inflammatory circuits and may be combined with chemotherapy or targeted agents into combinatorial regimens [189, 190].

A major critical aspect of senomorphic therapy is the temporal heterogeneity of the SASP. Early SASP responses may include immunosuppressive and pro‐fibrotic mediators such as TGF‐β, VEGF, and ECM proteins; these provide tissue protection but can also transiently suppress immune surveillance [191]. If senescence is unresolved, the SASP becomes largely represented by an inflammatory and degenerative profile of IL‐6, IL‐8, IL‐1β, and MMPs that promote senescence, foster chronic inflammation, and impair tissue function [191, 192]. Finally, when and how to modulate the SASP, such as too early may interfere with tissue repair and too late risks aggravating inflammation and tumor progression [190].

Senomorphics are being increasingly designed to complement senolytics in such multifaceted therapeutic strategies. For instance, the transient senomorphic treatment could suppress the SASP‐induced toxicity before the senolytics are administered to clear the accumulated senescent cells [193]. On the contrary, the post‐senolytic senomorphic interventions might help limit SASPs release from either residual or neighboring cells. This complementary approach finds support in preclinical studies addressing chemotherapy‐induced senescence, metabolic disorders, and neuroinflammation [190, 191, 192, 193].

Preclinical studies have demonstrated that pharmacological modulation of pathways involved in SASP regulation, including JAK‐STAT, NF‐κB, and mTOR signaling, can attenuate inflammatory SASP components and improve tissue homeostasis in experimental systems [4]. However, much of the current evidence derives from preclinical studies, and the long‐term efficacy and safety of senomorphic interventions in humans remain areas of ongoing investigation.

Importantly, the safety profile of senomorphic agents is likely to depend on the specific signaling pathway targeted, as well as the dose, duration of treatment, and tissue type. For example, inhibition of pathways such as JAK‐STAT or mTOR may influence immune regulation, metabolic processes, and tissue repair mechanisms [188]. Consequently, prolonged suppression of SASP signaling could potentially affect host defense responses, wound‐healing processes, or immune homeostasis, particularly in older individuals or patients undergoing intensive therapy [155, 194]. These considerations highlight the need for careful evaluation of condition‐specific risks and benefits when developing senomorphic therapies (Figure 5).

Rather than representing universally safer alternatives, senomorphic and senolytic approaches may offer complementary therapeutic strategies [169]. In some cases, transient SASP modulation may help limit chronic inflammation and tissue damage, whereas in others, selective elimination of persistent senescent cells through senolytic therapies may be required to prevent long‐term tumor‐promoting effects [169]. Determining the optimal strategy will likely depend on the condition, timing of intervention, and the extent of senescent cell accumulation [169].

Because SASP changes with tissue, stimulus, and stage of senescence, precision targeting is now going to be the focus of future senomorphic therapies. Antibody‐drug conjugates and AI‐guided drug discovery are in pursuit of refining the selective suppression of the deleterious components of SASP while preserving its beneficial paracrine functions [195]. Innovations such as these may enable condition‐specific modulation of senescence, allowing sustained control of chronic inflammation and age‐associated dysfunction without the systemic risks associated with global senescent cell elimination (Table 3).

**TABLE 3:** Senotherapeutic strategies targeting SASP: condition‐specific evidence, models, and safety considerations.

Agent	Mechanism	Model type	Cancer type	Senescence trigger	Key outcome	Clinical status	References
Senolytic							
Navitoclax (ABT‐263)	BCL‐2 Inhibition	Xenograft, in vitro	Breast lymphoma	TIS (chemotherapy)	Eliminates senescent cells and reduces SASP; delays tumor recurrence in xenograft models	Clinical trials; thrombocytopenia	[171]
Obatoclax	BH3 mimetic; BCL‐2 Inhibition	in vitro, xenograft	TNBC, glioma	TIS (drug‐induced)	Enhances sensitivity to targeted therapies (e.g., bromodomain inhibitors) and promotes apoptosis in preclinical models	Early clinical trials; neurotoxicity reported	[172]
Dasatinib + Quercetin	Multi‐kinase + flavonoid	In vitro, mouse models	Fibroblasts, multiple tumors	TIS/aging	Broad senolytic activity across cell types; improves tissue function in preclinical models	Early clinical evaluation; mild toxicity	[173, 177]
Fisetin	—	Mouse models, in vitro	Multiple	TIS/aging	Exhibits partial senolytic activity; reduces senescence‐associated phenotypes and induces apoptosis in preclinical systems	Under investigation; low toxicity reported	[174]
Procyanidin C1	‐‐	In vitro, xenograft	Prostate cancer	TIS	Induces apoptosis at high doses and suppresses SASP at low doses; associated with reduced tumor growth and chemoresistance in preclinical models	Preclinical investigation ongoing	[170]
Ouabain	Na^+^/K^+^‐ATPase targeting	In vitro	Multiple	TIS	Promotes selective elimination of senescent cells via Na^+^/K^+^‐ATPase targeting in cell‐based models	Not clinically used for senescence; cardiotoxicity concerns	[171]
FOXO4‐DRI peptide	FOXO4–p53 disruption	Mouse models, in vitro	Multiple	TIS/Aging	Induces apoptosis selectively in senescent cells by disrupting FOXO4–p53 interaction; improves tissue function in preclinical studies	Preclinical investigation ongoing	[171]
Senomorphics							
Rapamycin	mTOR inhibition	In vitro, mouse models	Multiple	TIS, OIS	Suppresses SASP and improves tissue homeostasis in preclinical systems	Clinically used; immunosuppression risk	[179]
Ruxolitinib	JAK/STAT blockade	Mouse models	Hematologic, solid tumors	TIS	Reduces inflammatory SASP and improves therapy response in preclinical models	Approved drugs; cytopenia risk	[182]
Roxadustat	—	Mouse models	Multiple	TIS	Suppresses SASP output and improves resilience to chemotherapy‐induced stress in preclinical models	Clinically approved (for anemia); off‐target effects under evaluation	[180]
ATI‐450/p38 inhibitors	p38 MAPK pathway inhibition	Mouse models	Breast cancer	TIS	Reduces SASP cytokines; limits metastasis	Preclinical; off‐target signaling effects	[181]

### 7.3 Novel Natural Senolytics and Senomorphics

Natural products, particularly polyphenols and flavonoids, represent a promising source of compounds capable of modulating senescence‐associated pathways, including stress response, apoptosis, and inflammation [196]. Preclinical studies have identified several phytochemicals with senolytic or senomorphic potential, such as quercetin and fisetin, which selectively target senescent cells and improve age‐related phenotypes, partly through inhibition of pro‐survival pathways (e.g., BCL‐2 family) and suppression of inflammatory SASP signaling [173, 174].

Other compounds, including curcumin, apigenin, and resveratrol, primarily act as senomorphics by inhibiting key SASP‐regulating pathways such as NF‐κB, mTOR, and JAK/STAT, thereby reducing inflammatory signaling and limiting the paracrine spread of senescence [188]. Additional phytochemicals such as Epigallocatechin gallate (EGCG), berberine, luteolin, kaempferol, and naringenin further contribute by regulating oxidative stress, mitochondrial function, and metabolic pathways [197, 198, 199, 200, 201].

Emerging evidence also highlights compounds like procyanidin C1 and wogonin derivatives, which may exhibit dose‐dependent dual activity, functioning as SASP suppressors at lower concentrations and senolytics at higher doses [175, 202]. Collectively, these findings suggest that natural compounds can target multiple nodes of the senescence network, offering a multimodal and potentially safer approach, although current evidence remains largely preclinical [197, 198, 199, 200, 201, 202] (Table 4).

**TABLE 4:** Natural senolytics and senomorphics with mechanisms and biological effects.

Compound	Category	Primary mechanism	Senolytic/senomorphic effects	References
Quercetin	Natural senolytic	Interferes with anti‐apoptotic BCL‐2 family signaling	Broad senolytic activity (often synergistic with Dasatinib); reduces SASP; improves age‐related phenotypes	[146]
Fisetin	Natural senolytic	Modulates anti‐apoptotic networks; antioxidant/apoptotic signaling	Promotes selective apoptosis of senescent cells; delays age‐ and cancer‐associated phenotypes	[146, 168]
Procyanidin C1	Dual (Senomorphic + Senolytic)	Low dose: SASP suppression; High dose: mitochondrial apoptosis induction	Reduces chemoresistance, tumor progression; eliminates senescent cells at high dose	[175]
Curcumin	Natural senomorphic (partial senolytic)	Inhibits NF‐κB, mTOR, MAPK; antioxidant activity	Suppresses SASP; reduces inflammatory cytokines; induces apoptosis in stressed/dysfunctional cells	[188]
Apigenin	Natural Senomorphic	Downregulates NF‐κB and JAK/STAT pathways; enhances autophagy	Reduces SASP secretion; lowers oxidative stress; limits paracrine senescence	[202]
Resveratrol	Natural Senomorphic	Activates SIRT1; improves mitochondrial function	Strong SASP suppression; restores metabolic fitness; anti‐aging effects	[126]
EGCG (Epigallocatechin gallate)	Senomorphic (context‐dependent senolytic)	AMPK activation; ROS scavenging	Reduces SASP; protects against oxidative stress; modulates mitochondrial stability	[199]
Berberine	Senomorphic	AMPK activation; anti‐inflammatory signaling	Reduces SASP; improves metabolic and oxidative stress responses	[197]
Luteolin	Natural Senomorphic	Inhibits NF‐κB, JAK–STAT, and SIRT1‐regulated pathways	Reduces UV‐induced, neuroinflammatory aging markers; suppresses SASP	[200]
Kaempferol	Senomorphic (partial senolytic)	AMPK activation; anti‐inflammatory signaling	Decreases SASP output; improves oxidative stress responses	[198]
Pterostilbene	Natural Senomorphic	Antioxidant, anti‐inflammatory signaling	Reduces oxidative/inflammatory SASP in skin, endothelial, liver senescence models	[196]
Rutin	Senomorphic	Modulates ATM‐related pathways	Interferes with early SASP development; bioavailability limitations	[196]
Hesperidin/hesperetin	Natural senomorphic	Regulates Nrf2, NF‐κB, FOXO pathways	Suppresses SASP; protective roles in bone and joint aging	[196]
Naringenin	Senomorphic	Modulates SIRT1–NF‐κB axis; improves mitochondrial function	Lowers SASP; improves redox balance; context‐dependent effects	[201]
Wogonin	Natural senomorphic	NF‐κB inhibition	Suppresses SASP; anti‐inflammatory effects	[202]
GL‐V9 (Wogonin derivative)	Natural senolytic	Alters lysosomal pH; increases ROS; triggers apoptosis	Selective elimination of senescent cells; strong senolytic activity	[202]

### 7.4 Biomarkers Associated to SASP

SASP factors generally emerge during damage‐induced, stress‐induced, or OIS [35]. However, confirmation of senescence  solely based on components of SASP remains difficult. Most SASP‐associated molecules are not unique to senescent cells but are also produced by immune cells, epithelial cells, and stromal populations upon infection, acute stress, or tissue injury [203]. This redundancy makes it difficult to interpret experimental readouts and again underlines the need for multimodal approaches that merge SASP detection with other markers of cell cycle arrest and chromatin remodeling [203].

Apart from their biomarker role in cellular senescence, components of SASP have recently been recognized as useful indicators of chronic inflammation and possible markers of therapeutic response or resistance [203]. High levels of cytokines of SASP, especially IL‐6, IL‐8, GM‐CSF, and several chemokines, are associated with increased inflammatory signaling, immune cell infiltration, and involvement of pro‐survival pathways, each reducing the effectiveness of chemotherapy, radiotherapy, and targeted therapeutics [204]. IL‐6/STAT3 and IL‐8/CXCR1‐2 signaling loops are also responsible of contributing to cancer stem‐like cell expansion and EMT and are associated with poor treatment response [205]. Many of these SASP components can be measured in tissue secretomes, plasma, or patient‐derived biofluids and are being investigated as non‐invasive biomarkers for monitoring TIS, predicting resistance, and guiding implementation of senolytic or senomorphic therapy [182, 183]. Profiling SASP factors, therefore, enhances mechanistic insights into TME cross‐talk and has the potential to improve patient stratification and personalized treatment.

Because many SASP components overlap with inflammatory mediators produced by non‐senescent cells, careful design of biomarker strategies is required. Several studies have emphasized the need for multi‐marker senescence panels and contextual validation [203]. Key considerations for developing reliable SASP‐based biomarkers are summarized in Table 5. Now, single‐cell transcriptomics and single‐cell proteomics enable the identification of individual senescent cells in heterogeneous tissues and accurately map SASP cytokines, chemokines, ECM enzymes, and growth factors [206]. Such high‐resolution profiling enables distinguishing proliferating, pre‐senescent, fully senescent, and escape‐like subpopulations, not possible from bulk‐level measurements [206].

**TABLE 5:** Key considerations for developing reliable SASP‐based biomarkers.

S/N	Do	Don't
1	Use multi‐marker panels rather than relying on a single cytokine or chemokine.	Do not rely on single inflammatory cytokines (e.g., IL‐6 or IL‐8) as standalone markers of senescence
2	Combine secreted SASP factors (e.g., IL‐6, IL‐8, GDF family members, MMPs) with cell‐state markers of senescence such as *p16* ^Ink4a^, *p21* ^CIP1^, DNA damage markers (γH2AX), or SA‐β‐gal activity.	Avoid interpreting elevated SASP factors without accounting for immune‐cell–derived inflammation.
3	Integrate circulating biomarkers with tissue‐based measurements (tumor biopsies, spatial transcriptomics, immunohistochemistry) to confirm the senescent cell origin.	Do not assume SASP signatures are uniform across different cancers or therapy contexts.
4	Validate biomarker panels across multiple tumor types, senescence triggers (OIS, TIS), and clinical cohorts	Avoid biomarker interpretation without considering the senescence trigger (therapy‐induced vs. oncogene‐induced).
5	Incorporate longitudinal sampling during therapy to capture dynamic changes in SASP composition.	Do not overlook clinical confounders such as infection, systemic inflammation, or treatment‐related immune activation.

These emerging integrative technologies now provide deeper mechanistic insight into how SASP programs originate and diversify across senescent states. Techniques like CITE‐seq merge transcriptomic signatures with surface protein expression to classify senescent cells with far greater fidelity, whereas scNMT‐seq has simultaneously profiled nucleosome positioning, DNA methylation, and gene expression to reveal how active epigenetic remodeling drives SASP heterogeneity [207, 208]. Spatial multi‐omics goes one step further by allowing precise localization of senescent cells and the characterization of spatially resolved SASP‐associated signaling within native tissue architecture and helps in understanding paracrine modes of senescence propagation, immune cell recruitment dynamics, and microenvironmental remodeling [208, 209]. These methods reveal the previously unrecognized subtypes of senescence states, including inflammatory, fibrotic, metabolic, and immune‐modulatory senescent states, each defined by unique SASP combinations which may differentially influence tumor progression or therapeutic response [210].

Computational frameworks further strengthen the application of these tools, including Seurat,an R package designed for scRNA‐seq data analysis for multimodal integration, scDAPP for senescence‐focused differential expression analysis, scmFormer for large‐scale multi‐omics modeling, and the curated SASP Atlas, which provides a proteomic reference for identifying SASP biomarkers [211, 212]. Collectively, these innovations support the creation of precision senescence biology in which specific SASP signatures are uncovered as diagnostic markers, predictors of therapy resistance, or targets for senolytic/senomorphic interventions. As single‐cell and spatial multi‐omics technologies continue to mature, they will feature centrally in the design of future therapeutic strategies aimed at selectively modulating or eliminating few senescent cell subsets while preserving beneficial senescence‐associated functions [212].

A major challenge in the clinical application of SASP‐associated biomarkers is distinguishing senescence‐specific secretory profiles from general inflammatory responses [213]. Many commonly studied SASP components, including IL‐6, IL‐8, TNF‐α, and CCL2, are not unique to senescent cells and may also be elevated during infection, tissue injury, autoimmune disorders, metabolic disease, or systemic inflammation [213]. Therefore, reliance on single circulating cytokines is insufficient to infer the presence of senescent cells or senescence‐driven pathology [213]. Instead, current evidence supports the use of integrated multi‐marker approaches combining soluble SASP factors with tissue‐associated senescence markers such as p16^Ink4a^, p21, SA‐β‐gal activity, DNA damage markers, extracellular vesicle signatures, and context‐specific histopathological assessment [214].

### 7.5 SASP‐Mediated Modulation of Immunotherapy and CAR‐T Responses

Senescent T cells have been found to accumulate during the course of aging and in cancer‐bearing individuals, thus contributing to an immunosuppressive microenvironment that may counteract the effectiveness of various immunotherapies. As a matter of fact, Kasamatsu et al., 2023 [215] reported that such senescent T cells disclose typical features of cellular aging, their SASP producing pro‐inflammatory cytokines, chemokines, and inhibitory molecules promoting T‐cell exhaustion and dysfunction [215]. These findings raise critical points regarding cancer immunotherapy, as senescent T‐cells may dominate the effector lymphocyte pool, weakening the capability of the immune system to mount a strong response against the tumor [216, 217]. In this regard, treatments for which efficient T‐cell responses are required such as checkpoint blockade, may function sub‐optimally, particularly in elderly patients or those with a high burden of senescent immune cells [217]. The presence of SASP in T‐cells would significantly impact the design and efficacy of CAR‐T therapy. In engineering CAR‐T cells, expansion and persistence are key to long‐term tumor control, although both can be markedly limited by senescence [218].

## 8 Limitations and Validation of SASP Biomarkers

For SASP‐associated biomarkers to achieve clinical utility in oncology, a structured validation framework is required. First, analytical validity must establish that biomarker assays are reproducible, sensitive, and technically robust across laboratories and sample types [214]. Second, biological specificity is essential to distinguish senescence‐associated secretory signals from non‐specific inflammatory responses caused by infection, tissue injury, or chronic inflammatory disease [213]. This requires integration of circulating SASP factors with tissue‐level senescence markers and longitudinal sampling strategies rather than reliance on isolated cytokines [213, 214].

Third, clinical validity must demonstrate reproducible associations between SASP biomarker profiles and clinically meaningful outcomes such as treatment response, TIS burden, recurrence risk, or survival across independent patient cohorts [194, 213]. Finally, clinical utility requires evidence that biomarker‐guided interventions improve patient outcomes, treatment stratification, toxicity prediction, or therapeutic decision‐making compared with current standard approaches [219]. At present, most proposed SASP biomarkers remain at the exploratory or preclinical validation stage, and substantial standardization efforts are still needed before routine clinical implementation [219].

## 9 Conclusion

Cellular senescence, and particularly the SASP, has emerged as a critical but highly condition‐dependent regulator of cancer. A central theme across the literature is the temporal and functional duality of SASP. In early or transient phases, typically within days following senescence induction, SASP can promote immune surveillance, reinforce growth arrest, and support therapy‐induced tumor clearance. In contrast, persistent SASP, which commonly develops under conditions of unresolved TIS, is associated with chronic inflammation, immunosuppression, ECM remodeling, and angiogenesis. Accumulating evidence from both preclinical and clinical studies indicates that sustained SASP signaling can promote tumor progression, facilitate therapeutic resistance, and contribute to an unfavorable TME. Importantly, these outcomes vary depending on the senescence trigger (e.g., OIS vs. TIS), cellular origin, tumor type, and microenvironmental conditions, underscoring the need for cautious interpretation of generalized claims.

The immunological consequences of SASP further highlight this complexity. While acute SASP can enhance anti‐tumor immunity and improve responses to chemotherapy, radiotherapy, and immunotherapy in certain models, chronic SASP frequently drives immunosuppressive networks, including recruitment of MDSCs and Tregs, thereby limiting therapeutic efficacy. In addition, senescence escape, observed in several experimental systems, may give rise to aggressive, stem‐like, and therapy‐resistant cell populations, partially shaped by prolonged SASP exposure. These observations collectively emphasize that the timing and duration of SASP signaling are critical determinants of therapeutic outcome, reinforcing the need for temporally informed intervention strategies.

Therapeutically, both senolytic and senomorphic approaches offer promising but condition‐dependent applications. Senolytics aim to eliminate persistent senescent cells and have demonstrated efficacy in preclinical models, although their clinical application remains constrained by on‐target toxicities and tissue‐specific effects. Senomorphics, by contrast, modulate SASP without removing senescent cells and may preserve beneficial aspects of transient senescence; however, their long‐term effects on immune function, tissue repair, and host defense require careful evaluation. Rather than representing universally preferable strategies, these approaches are likely to be complementary, with optimal application dependent on disease, timing, and senescence burden.

From a translational perspective, SASP components hold potential as dynamic biomarkers, but their clinical utility requires multi‐marker, cell‐state anchored approaches that integrate circulating factors with tissue‐based indicators of senescence. Given that many SASP factors overlap with general inflammatory mediators, contextual interpretation and longitudinal monitoring are essential to avoid misclassification. Advances in single‐cell, spatial, and multi‐omics technologies are beginning to enable more precise mapping of senescent cell states and SASP heterogeneity within the TME, offering new opportunities for biomarker development and therapeutic targeting. Although senescence‐targeted strategies, including senolytics and senomorphics, show strong potential in preclinical cancer models, their clinical application remains limited. Key challenges include tissue‐specific effects, incomplete understanding of SASP heterogeneity, biomarker inconsistency, and potential disruption of beneficial senescence‐associated immune surveillance. Therefore, these approaches should currently be considered investigational rather than established clinical strategies.

In summary, the integration of cellular senescence into cancer management will depend on the ability to distinguish beneficial, transient senescence from persistent, pathological states and to align therapeutic interventions accordingly. Future progress will require condition‐specific senotherapeutic strategies, robust biomarker frameworks, and a deeper mechanistic understanding of SASP dynamics across tumor types and treatment settings. Such advances may ultimately enable the selective harnessing of senescence‐associated tumor‐suppressive mechanisms while minimizing the deleterious consequences of chronic SASP, thereby improving clinical outcomes and advancing treatment approaches in precision oncology.

## Author Contributions

**Sneha Pallatt:** writing – original draft, conceptualization, writing – review and editing. **Sibin Nambidi:** writing – original draft, writing – review and editing. **Antara Banerjee:** supervision, conceptualization, writing – original draft, writing – review and editing. **Surajit Pathak:** conceptualization, writing – original draft, writing – review and editing, supervision.

## Funding

The authors have nothing to report.

## Conflicts of Interest

The authors declare no conflicts of interest.

## Data Availability

Data sharing not applicable to this article as no datasets were generated or analysed during the current study.
